# A cluster-randomized, placebo-controlled trial to evaluate the efficacy of a spatial repellent (Mosquito Shield™) against *Aedes*-borne virus infection among children ≥ 4–16 years of age in the Gampaha District, Sri Lanka: study protocol (the AEGIS program)

**DOI:** 10.1186/s13063-022-06998-z

**Published:** 2023-01-04

**Authors:** Hasitha Tissera, D. S. Anoja F. Dheerasinghe, Neelika Malavige, H. Asita de Silva, Amy C. Morrison, Thomas W. Scott, Robert C. Reiner, John P. Grieco, Nicole L. Achee

**Affiliations:** 1 Epidemiology Unit, Ministry of Health, Colombo, Sri Lanka; 2 National Dengue Control Unit (NDCU), Ministry of Health, Colombo, Sri Lanka; 3 Department of Immunology and Molecular Medicine, Faculty of Medical Sciences, University of Sri Jayewardenepura, Nugegoda, Sri Lanka; 4 Clinical Trials Unit, Faculty of Medicine, University of Kelaniya, Kelaniya, Sri Lanka; 5 University of California Davis, Davis, CA USA; 6 University of Washington, Seattle, WA USA; 7 Department of Biological Sciences, Eck Institute for Global Health, University of Notre Dame, 243 Galvin Life Science Center, Notre Dame, IN 46556 USA

**Keywords:** Spatial repellent, Dengue, *Aedes*-borne Viruses, Transfluthrin, Incidence, Cluster-randomized controlled trial, Sri Lanka

## Abstract

**Background:**

Spatial repellents (SRs) have been widely used for prevention of mosquito bites, but their efficacy in reducing *Aedes*-borne viruses (ABV) has not been tested rigorously at large scale in Asia. To address this knowledge gap, a trial to evaluate the efficacy of Mosquito Shield™, a transfluthrin SR, was developed in Gampaha District of Sri Lanka across three Medical Officer of Health areas; i.e., Negombo, Wattala, and Kelaniya.

**Methods:**

This trial is a cluster-randomized, placebo-controlled, double-blinded clinical trial. A total of ~14,430 subjects aged ≥ 6 months in 30 clusters (15 intervention, 15 placebo) from ~3900 households (HH) will be randomly selected for enrolment into a “febrile surveillance cohort.” A subset of the surveillance cohort, ~3570 subjects aged ≥4–16 years that test seronegative (naïve) or are serologically positive for a previous single dengue virus (DENV) infection (monotypic) at baseline sampling, will be enrolled into a “longitudinal cohort” for measuring DENV infection based on laboratory-confirmed seroconversion during the trial. Persons identified positive for antibodies against multiple DENV serotypes (multitypic) at baseline will be monitored for secondary analyses.

Active ABV disease will be assessed using an enhanced passive surveillance system with case ascertainment performed in designated healthcare facilities. Serum samples will be taken from longitudinal cohort subjects within 1–2 weeks of when intervention is first deployed (T0) with additional samples taken ~12 (T1) and ~24 months (T2) from baseline sampling. DENV seroconversion and ABV active disease rates from baseline (pre-intervention) and follow-up (post-intervention) samples will be compared between intervention and placebo clusters. Participating houses will be monitored entomologically (indoor adult *Aedes aegypti* population densities and adult female blood fed status) within 3 months before intervention deployment and monthly during the intervention phase. Entomological surveys will monitor indoor adult *Ae. aegypti* population densities and blood fed status. Dengue incidence in each cohort will be estimated and compared to determine the public health benefit of using an SR. Entomological parameters will be measured to determine if there are entomological correlates of SR efficacy that may be useful for the evaluation of new SR products.

**Discussion:**

The trial will serve as an efficacy assessment of SR products in South Asia. Results will be submitted to the World Health Organization Vector Control Advisory Group for assessment of public health value towards an endorsement to recommend inclusion of SRs in ABV control programs.

**Trial registration:**

Sri Lanka Clinical Trial Registry SLCTR/2022/018. Registered on July 1, 2022.

ClinicalTrials.govNCT05452447. Registered on July 11, 2022.

The Universal Trial Number is U1111-1275-3055.

## Administrative information

Note: the numbers in curly brackets in this protocol refer to SPIRIT checklist item numbers. The order of the items has been modified to group similar items (see http://www.equator-network.org/reporting-guidelines/spirit-2013-statement-defining-standard-protocol-items-for-clinical-trials/).

**Table:** 

Title {1}	A cluster randomized, placebo control trial to evaluate the efficacy of a spatial repellent (Mosquito Shield™) against *Aedes*-borne virus infection among children ≥ 4–16 years of age in the Gampaha District, Sri Lanka: study protocol (the AEGIS program)
Trial registration {2a and 2b}.	Sri Lanka Clinical Trial Registry SLCTR/2022/018. Registered July 1, 2022. https://slctr.lk/trials/slctr-2022-018 ClinicalTrials.gov NCT05452447. Registered July 11, 2022. https://clinicaltrials.gov/ct2/show/record/NCT05452447?cond=dengue&cntry=LK&draw=2&rank=4 The Universal Trial Number is U1111-1275-3055.
Protocol version {3}	Version 9.1 - February 3, 2022
Funding {4}	The project under which the data will be gathered, “Advancing Evidence for the Global Implementation of Spatial Repellents (AEGIS),” is possible thanks to Unitaid funding and support. Unitaid is a global health agency engaged in finding innovative solutions to prevent, diagnose, and treat diseases more quickly, effectively, and for affordable prices, in low- and middle-income countries. Unitaid’s work includes funding initiatives to address major diseases such as HIV/AIDS, malaria, and tuberculosis, as well as HIV co-infections and co-morbidities such as cervical cancer and hepatitis C, and cross-cutting areas, such as fever management. Unitaid is now applying its expertise to address challenges in advancing new therapies and diagnostics for the COVID-19 pandemic, serving as a key member of the Access to COVID Tools Accelerator. Unitaid is hosted by the World Health Organization. Additionally, SC Johnson, A Family Company, will use internal company financial resources for the development, manufacturing, delivery, and shipment of the intervention used in the study.
Author details {5a}	HT: Epidemiology Unit, Ministry of Health, Sri Lanka DSAFD: National Dengue Control Unit, Ministry of Health, Sri Lanka NM: Department of Immunology and Molecular Medicine, Faculty of Medical Sciences, University of Sri Jayewardenepura, Sri Lanka HAS: Clinical Trials Unit, Faculty of Medicine, University of Kelaniya, Sri Lanka AM, TS: University of California Davis RR: University of Washington JPG, NLA: University of Notre Dame, Notre Dame, IN 46556, USA
Name and contact information for the trial sponsor {5b}	Dr. John P. Grieco Lead Principal Investigator, Advancing Spatial Repellents for Vector-Borne Disease Control Eck Institute for Global Health University of Notre Dame 243 Galvin Life Science Notre Dame, IN 46556 jgrieco@nd.edu 574.631.7572
Role of sponsor {5c}	As study sponsor, the University of Notre Dame (UND) participated in study design, management, analysis, data interpretation, and manuscript development As funder, Unitaid had no role in the design of the study and collection, analysis, and interpretation of data and in the writing of the manuscript.

## Introduction

### Background and rationale {6a}

Dengue viruses (DENVs) are a medically important arthropod-borne pathogens worldwide, with transmission occurring in most tropical and subtropical regions. An estimated 390 million infections occur yearly [1]. Although there are considerable ongoing efforts to further develop and implement a dengue vaccine [2, 3], vector control remains the primary option for reducing DENV transmission and the dengue disease burden. Yellow fever virus and the recent emergence of *Aedes*-borne Zika virus (ZIKV) and Chikungunya virus (CHIKV) further highlight the growing need for a range of novel and effective vector control tools, which includes spatial repellents (SRs). Herein we use spatial repellency as a general term to refer to a range of insect behaviors caused by airborne chemicals including movement away from a chemical stimulus, and/or interference with host detection (attraction-inhibition), and/or blood feeding response. Any one or combination of these behaviors can reduce the probability of contact between people and virus-infected mosquito vectors, and thus pathogen transmission.

A recent study in Iquitos, Peru, demonstrated a significant protective effect of a SR product against *Aedes*-borne virus (ABV) transmission with a statistically significant reduction in the density of indoor female *Aedes aegypti* mosquitoes [4]. The use of a SR to demonstrate a reduction in ABV transmission has not been evaluated at large scale in Asia. If effective, it could enhance vector control practices in the region and/or worldwide by complementing current disease prevention strategies.

This trial will be carried out in Sri Lanka, which has a well-established infrastructure for studying urban dengue. The trial is a component of a multi-center research program testing the same SR product, Mosquito Shield™, in Mali and Kenya to evaluate its impact against malaria, another mosquito-borne disease [https://aegis.nd.edu/]. The study, therefore, is designed to generate rigorous evidence, documenting and evaluating the impact of SR products on human arthropod-borne pathogen infection rates, to be considered and used by academia, industry, and public health key stakeholders at the global, regional, national, and/or local level. Results will contribute to assessments by the World Health Organization (WHO) regarding the recommendation of SR products for inclusion in disease control programs.

Over 3.9 billion people in 129 countries are at risk of DENV infection annually. Recent estimates are 390 million DENV infections per year (95% credible interval 284–528 million), of which 96 million (67–136 million) manifest clinically (with any severity of disease) [1]. Beyond the direct impact on afflicted individuals, urban dengue epidemics overwhelm public health systems and destabilize societies [5]. Incidence rates have steadily increased since the 1950s [6] with severe economic consequences for endemic countries that negatively impact economic development and contribute to political instability. A mosquito-borne arbovirus belonging to the family Flaviviridae, DENV is capable of causing dengue fever, dengue hemorrhagic fever (DHF), and dengue shock syndrome (DSS) [7]. The predominant urban vector of DENV, *Aedes aegypti* (L) (Diptera: Culicidae), is a peri-domestic, day-biting mosquito species found throughout the tropical and semi-tropical world. *Aedes aegypti* larvae inhabit small, localized habitats (i.e., artificial containers, tires, water storage containers, disposal plastic containers) and thus are difficult to control before adult mosquitoes emerge [8, 9]. Adult stages are commonly found in secluded areas of a dwelling, such as resting underneath beds or on clothes in the lower portions of closets. *Ae. aegypti* also transmit other arboviral infections, including Zika, Chikungunya, and urban yellow fever.

Presently, dengue prevention relies on vector control. It is focused on routine larval source management (i.e., elimination or treatment of larval habitats to prevent adult development) and reactive space spray or other adult-focused interventions to reduce adult population densities [10–12]. Although a mainstay of most *Ae. aegypti* control programs [13], spraying is expensive and unless done repeatedly inside houses it is often ineffective because *Ae. aegypti* rest in places that are not penetrated by outdoor sprays; e.g., closets inside houses [14]. Consequently, as the scope of dengue continues to grow, new tools will be needed to compliment the limited number of available interventions and/or further optimization current products required to meet public health demands. This includes new paradigms for *Ae. aegypti* control targeting adult mosquitoes [15]. A scalable intervention, like SRs that can be easily deployed or provided through a consumer market, could be used to target adult female *Ae. aegypti* inside homes. This could help to fill current gaps in intervention programs by meeting the challenges of intervention coverage across large and complex urban environments.

Spatial repellency as defined above can include movement away from a chemical stimulus, interference with host detection (attraction-inhibition) and/or the host feeding response [16]. Spatial repellency can be measured and distinguished from other chemical actions, including contact irritancy and toxicity, in laboratory studies [17, 18]. SRs have been shown to effect (behavior change) against insecticide-resistant mosquito populations and have the potential to limit the spread and/or emergence of insecticide resistance alleles due to reduced selection pressure when there is a non-lethal effect. SRs could be offered as stand-alone tools where no other interventions are currently in use or, most likely, combined with existing interventions to augment the efficacy of other tools, i.e., a combination strategy. SRs drive mosquitoes away from a treated space. Toxins kill mosquitoes, and irritants are compounds that rely on contact between the mosquito and a treated surface to prevent resting. Many compounds exhibit two or more modes of action, but they can be distinguished by the concentration or dose needed to achieve them [19]. Spatial repellency occurs at low vapor phase concentration, contact irritancy requires higher doses, and killing requires absorption of still higher doses.

SRs have been demonstrated to prevent human-vector contact using vapor-active pyrethroids. Effected mosquitoes include major genera (e.g., *Anopheles* spp. and *Aedes* spp.) and a range of insecticide susceptibility statuses. In semi-field conditions, Phase II (experimentally controlled conditions) mosquitoes in natural environments in Belize [20] and Thailand [19] did not enter experimental huts that contained a SR source. Kawada et al. [21–23] showed indoor and outdoor protection (up to 90% bite reduction) against *Anopheles* spp. and *Culex* spp. when using metofluthrin-impregnated paper strips as compared to untreated sites in field conditions in Lombok, Indonesia. Evaluations of transfluthrin showed up to 90% bite protection from *Anopheles* spp. inside houses of Dar es Salaam, Tanzania, compared to controls after volatizing using a kerosene oil lamp [24]. Outdoor field trials in North America demonstrated paper emanators impregnated with metofluthrin reduced landing rates of *Aedes vexans* by up to 95% compared to pre-treatment conditions [25] and *Ae. canadensis* by 85–100% compared to untreated controls [26]. Both species are nuisance biters and incriminated in transmission of West Nile Virus. Assessments of a passive metofluthrin emanator in a randomized trial of 200 houses in Mexico demonstrated significant reductions in indoor *Ae. aegypti* female abundance (58%) and females that contained blood meals (57.2%) as compared to homes without the SR [27]. In addition, experimental hut studies in Iquitos, Peru, demonstrated XX% reduction in repellency (SOURCE).

There is evidence from Phase III studies that SR products can impact vector-borne pathogen transmission. A recent large-scale clinical trial in Iquitos, Peru, using a passive transfluthrin emanator, demonstrated a significant protect effect (34%) against ABV infection in treated clusters receiving the active intervention compared to placebo clusters and a significant reduction in adult *Ae. aegypti* female indoor abundance (28%) and blood fed rate (12%) compared to baseline [4]. A proof-of-principle study in Sumba Island, Indonesia, showed that communities (village clusters) with high coverage (90%) of a metofluthrin mosquito coil had 60% reduced malaria transmission and had lower biting pressure from anopheline vector mosquitoes [28]. In a large-scale cluster-randomized control trial (cRCT), Syafruddin et al. [29] demonstrated the ability of a transfluthrin passive emanator to decrease new malaria infections by 60% in study populations residing in moderate- to high-risk clusters. Replication and extension of these studies will be the core of the work outlined in the current protocol.

Over the past decade, formal national and international meetings have been convened to bring together academics, industry, and global public health experts, including representatives from the WHO and Vector Control Advisory Group (VCAG), to discuss the role of SR products in the reduction of arthropod-borne diseases such as malaria and dengue. A key aspect of these meetings and subsequent efforts was to establish a critical path of development (CPD) for SR products based on expert advice. This includes measures related to scientific, regulatory, and social parameters. In part, the criteria developed outline the endpoints of a SR target product profile, i.e., optimum product characteristics. A major hurdle in the CPD for SR products has been the generation of sufficient epidemiological data demonstrating significant protective efficacy (PE) to influence policy makers to confidently recommend the incorporation of SRs into current multi-lateral disease control programs. Realizing this vision demands credible demonstration of PE against new human infections and estimates of SR PE based on vector bionomics, intervention coverage, and/or virus transmission rates which can be used to define optimum patterns of use.

To address this issue, we adapted the cRCT study design implemented in Iquitos, Peru, by Morrison et al. [4]. Our design uses similar epidemiological and entomological endpoints as in Peru to provide evidence from a second trial required by WHO VCAG to assess the public health value of SRs in different ecological settings. The use of a standardized core protocol for the Sri Lanka trial and Peru trial will facilitate a meta-analysis of the primary outcome (SR PE using seroconversion as indicator). Our goal is to generate data that can be reviewed by WHO to recommend (or not) the use of SR products in ABV disease prevention and control. Formal recognition of a SR paradigm for disease prevention will (1) facilitate the discovery and development of such products by industry manufacturers, (2) drive the expansion of new mosquito control approaches, and (3) allow for the recommendation of existing SR tools that can provide substantial impact on virus transmission worldwide; something which is vital in the fight against arthropod-borne diseases. This translates to protection against a broad range of diseases and virus vectors that could extend beyond dengue-endemic countries.

### Objectives {7}

The primary objective of this trial is to demonstrate and quantify the PE of Mosquito Shield™ in reducing the incidence of ABV infection, as measured by detection of serotype specific neutralizing antibodies in children ≥4–16 years of age whose serostatus at baseline is seronegative for DENV indicating they have not had a prior DENV infection or monotypic for DENV indicating prior infection with a single DENV serotype. Testing for ZIKV and CHIKV seroconversion will depend on circulation history/detection in the study area during the trial period.

The “longitudinal cohort” will receive standard entomological surveillance and control procedures by the local Ministry of Health. Seroconversion rates will be compared between intervention and control clusters, which will be under routine standard of care practices for the study area. The null hypothesis (H0) is that there is no difference in ABV infection between intervention and placebo-controlled arms (seroconversion odds ratio (OR) between SR and placebo is <1; expected OR is 70% or PE is 30%).

Secondary objectives will address key issues related to the optimization and application of SR products for public health and confirm the range of contexts within which SR PE can be achieved.

#### Secondary objectives are:

1. Estimating PE against aggregated DENV (multitypics), ZIKV, and CHIKV seroconversion.
2. Estimating the reduction in ABV disease in trial clusters.
3. Estimating the entomological correlates of reduced ABV infection based on measures of adult female *Ae. aegypti* indoor density and blood fed rate (proxy for human-biting rate).
4. Quantifying PE in relation to Mosquito Shield™ coverage and seasonal effects.
5. Quantifying the total number of infections averted using Mosquito Shield™.

The secondary outcome of ABV disease incidence will be assessed by passive monitoring of active febrile cases during the trial in order to compare results from intervention and control clusters. Participants followed for disease will be the “febrile surveillance cohort.” Persons positive for antibodies against multiple DENV serotypes (multitypic) at baseline will also be recruited for secondary analyses. Testing for ZIKV or CHIKV infection will be dependent on circulation history/detection in study area during trial period.

#### Tertiary objectives are:

1. To evaluate the safety of the SR product in human subjects.
2. Evaluate potential diversionary effects of the SR intervention to surrounding homes.

### Trial design {8}

This study will be a prospective, participant and observer-blinded, cRCT in the Gampaha District of Sri Lanka to measure the impact of a SR product on new ABV virus infections, consisting of a total of a 24-month follow-up with intervention. A total of 30 clusters per arm will be assigned; 15 SR and 15 placebo. Clusters of households (HH) will be selected from three Medical Officer of Health (MOH) areas in the district of Gampaha: Negombo, Wattala, Kelaniya. Each cluster will contain approximately 110–120 residents testing negative for antibodies against DENV (seronegative) or positive to a single DENV infection (monotypic).

DENV infection in trial participants will be assessed passively by monitoring febrile persons for acute Dengue illness (secondary outcome—“febrile surveillance cohort,” persons ≥6 months) and actively through serologic testing of scheduled longitudinal blood samples (primary outcome—longitudinal cohort, persons ≥ 4–16 years). Seroconversion in follow-up samples (post-intervention) and active disease rates will be compared between SR intervention and placebo (control) clusters. As noted above, detection of ZIKV and CHIKV infection at baseline and during the intervention phase of the trial will be dependent on circulation history/detection in the study area during the study period.

All participating HHs in each cluster will be monitored entomologically for adult *Aedes aegypti* and *Aedes albopictus* before initial deployment of the SR intervention and monthly after the intervention is in place. Entomological surveys will include monitoring of indoor *Ae. aegypti and Ae. albopictus* adult female mosquito population densities and blood fed status.

## Methods: participants, interventions, and outcomes

### Study setting {9}

The trial will be carried out in Sri Lanka, where dengue is endemic. Epidemic dengue was first recognized in Colombo, the capital of Sri Lanka, in 1965–1966 [30]. Outpatient, clinic-based surveillance at Colombo’s Lady Ridgeway Children’s Hospital (1980–1984) reported dengue accounted for 16% of acute febrile illness, of which 66% were recurrent dengue cases. A 1980–1985 school-based study found a baseline DENV seroprevalence of 50% in Colombo and a 6-month dengue incidence of 15.6%, of which 37% were secondary cases. In the early 1980s, severe dengue was rare in Sri Lanka; <10 reported cases were DHF [30]. Since 1989, however, many cases of DHF have been reported from the heavily urbanized western coastal belt of Sri Lanka, which includes Colombo [31], and cases have recently been reported elsewhere in the country. Epidemic CHIKV was reported in the Indian subcontinent in 2005, first in India and then in neighboring Sri Lanka (population ~ 20 million) in November 2006 [32]. After a 40-year hiatus [33], >37,000 suspected cases of CHIKV were reported in densely populated regions in the north, east, and western coastal belt of Sri Lanka in 2007 and a similar number in 2008 [34, 35]. Zika has not been reported from Sri Lanka, but surveillance efforts are currently present to detect its introduction. A complicating factor has been the observation that diagnosis in serum or plasma is very time sensitive with only days to effectively isolate the virus, but the virus persists in urine and other body fluids for longer periods of time out to weeks and as long as 6 months in semen. A further complicating factor is often CHIKV patients can be afebrile, but present with dengue-like rashes.

**Fig. 1:**
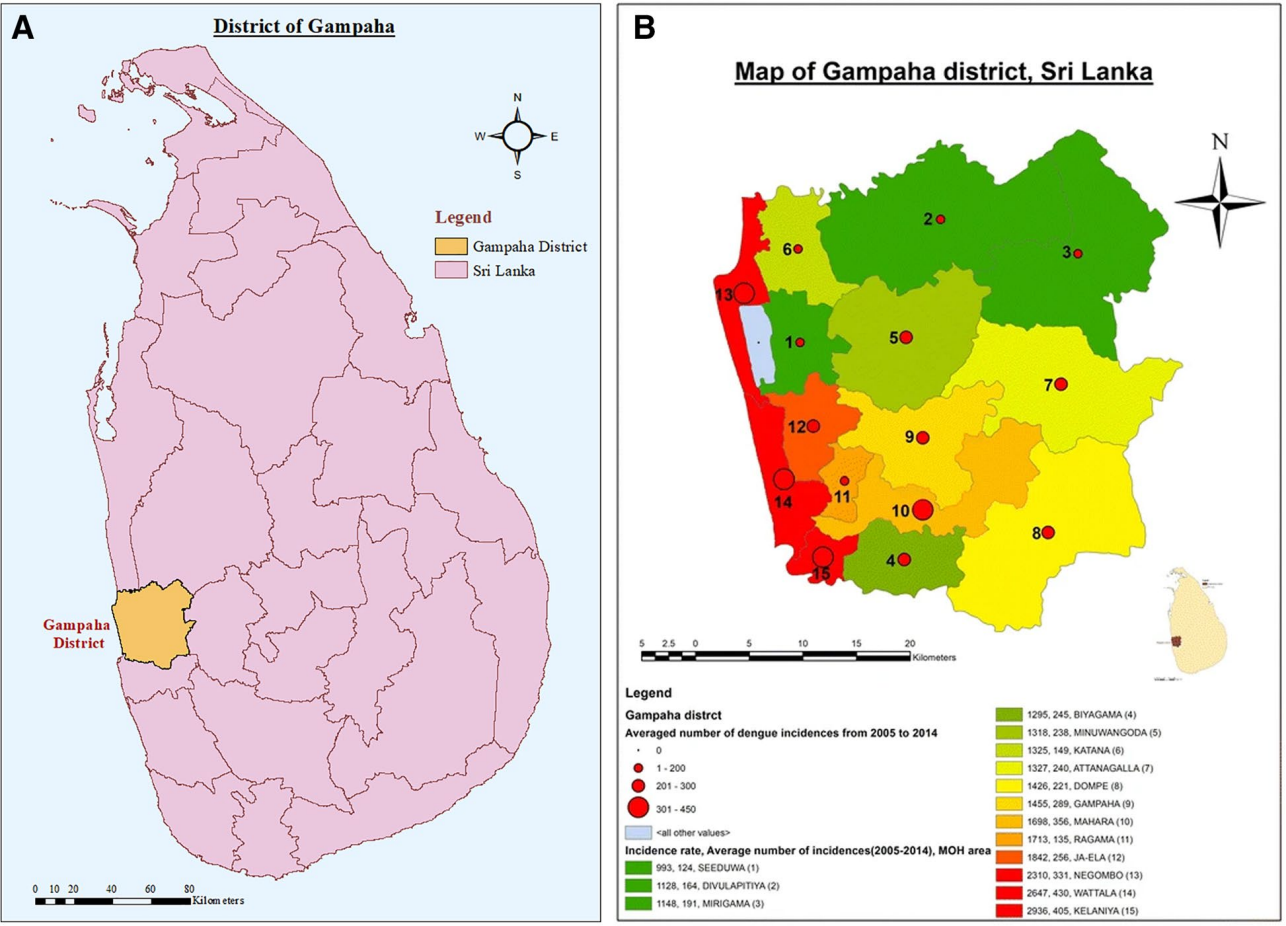
Gampaha District (**A**)^1^ and Negombo (13), Wattala (14), and Kelaniya (15) proposed MOH study areas (**B**)^2^. ^1^Withanage, G. P., Hapuarachchi, H. C., Viswakula, S. D., Gunawardena, Y. N. S., & Hapugoda, M. (2020). Entomological surveillance with viral tracking demonstrates a migrated viral strain caused Dengue epidemic in July, 2017 in Sri Lanka. PloS one, 15(5), e0231408. ^2^Withanage, G.P., Viswakula, S.D., Nilmini Silva Gunawardena, Y.I. et al. A forecasting model for Dengue incidence in the District of Gampaha, Sri Lanka. Parasites Vectors 11, 262 (2018)

All clusters will occur in 3 MOH areas in the district of Gampaha: Negombo, Wattala, and Kelaniya (Fig. 1). These areas were selected due to their similar epidemiology, ecology, proximity to each other, and ease of access to the implementers of the trial.

Previous census information for the 3 MOH areas selected within Gampaha indicates a relatively constant age distribution among children and adolescents: 7.59% are 0–4, 7.80% are 5–9, and 7.39% are 10–14. Procedures requiring blood samples will involve males and females limited to ≥ 4–16 years of age. Children are a significant risk group and represent the population with the highest number of susceptible individuals, thus their inclusion is essential to assess local DENV transmission dynamics. We will exclude younger children because of the inherent concerns and difficulties obtaining blood from small children. Assuming that within an age bin that the age-specific distributions are uniform, we estimated the percent of the population of Gampaha between ≥ 4 and 16 years with zero or one DENV infections. For children ages ≥ 4–16 years and taking population weighted averages, we estimated 58% are seronegative and 33% are monotypic for DENV infection.

Pregnant women will be present on the study blocks and allowed to participate in all aspects of the study because they represent a group at risk for dengue. Little information is available on the effects of DENV or CHIKV infection on fetuses and the procedures for our study do not represent additional risk to mothers. ZIKV infection represents a risk for fetuses, but our study procedures do not represent additional risk to mothers and might contribute to their protection. Pregnant women will be provided information on Zika and counseled to take as many personal protection precautions as possible. The fact that they could be in a negative cluster will be emphasized and additional protections will be recommended.

### Eligibility criteria {10}

The inclusion and exclusion criteria for all studies activities are listed in Tables 1, 2, 3, and 4 below. Screening for study eligibility will occur for four major study activities: (1) febrile surveillance (clinical disease), (2) longitudinal seroconversion follow-up (primary outcome), (3) Mosquito Shield™ household application inside homes, and (4) entomological surveys.

**Table 1:** Inclusion and exclusion criteria for febrile surveillance (clinical disease)

Febrile Surveillance (clinical disease)	
**Inclusion criteria**	**Exclusion criteria**
**Household level (written consent)**	
● Adult head of household agrees to census, health visits, and logging resident symptoms when febrile (or in the case of suspected Zika in the absence of fever, presenting with rash, arthralgia, arthritis, or non-purulent conjunctivitis). ● Individuals spend a minimum of 4 h per week during the daytime hours or sleep in the house.	● Adult head of household does not agree to census, health visits, or logging symptoms of residents. ● Households where study personnel identify a security risk (i.e., site where drugs are sold, residents are always drunk or hostile). ● Sites where no residents spend time during the day (i.e., work 7 days a week outside the home).
**Individual level (written consent)**	
● ≥ 6 months of age. ● Fever at the time of presentation or report of feverishness within the previous 24 h or presenting with a rash, arthralgia, arthritis, or non-purulent conjunctivitis (suspicion of ZIKV determined by project physician). ● Individual who spends a minimum of 4 h per week within the household or sleeps in the house.	● <6 months of age. ● No fever at time of presentation or report of feverishness within the previous 24 h or not reporting with a rash, arthralgia, arthritis, or non-purulent conjunctivitis. ● Individuals who have spent less than 4 h in the household during the week prior to illness.

**Table 2:** Inclusion and exclusion criteria for longitudinal seroconversion follow-up (to include baseline serostatus survey)

Longitudinal seroconversion follow-up (to include baseline serostatus survey) Individual level	
Inclusion criteria	Exclusion criteria
● ≥4 – 16 years of age ● Plans to stay in residence and/or study area for a minimum of 24 months ● Resident of household or frequent visitor (~20% of day hours in house/months)	● <4 and >16 years of age ● Plans to leave residence and/or study area within next 24 months ● Temporary visitor to household (<20% of day hours in house/months)

**Table 3:** Inclusion and exclusion criteria for Mosquito Shield™ household application

Mosquito Shield™ Household Application – Household Level (Written consent)	
Inclusion criteria	Exclusion criteria
● Adult head of household agrees to have intervention applied inside the home and to provide access to team member at 4-week intervals to change products. ● Properties where study personnel do not identify a security risk (i.e., site where drugs are sold, residents are always drunk or hostile).	● Adult head of household does not agree to Mosquito Shield™ deployment or study team access. ● Properties where study personnel identify a security risk (i.e., site where drugs are sold, residents are always drunk or hostile).

**Table 4:** Inclusion and exclusion criteria for entomological surveys

Entomological Surveys – Household Level (Written consent)	
Inclusion criteria	Exclusion criteria
● Adult head of household agrees to entomological surveys. ● Properties where study personnel do not identify a security risk (i.e., site where drugs are sold, residents are always drunk or hostile).	● Adult head of household does not agree to entomological surveys. ● Properties where study personnel identify a security risk (i.e., site where drugs are sold, residents are always drunk or hostile).

Every resident of a HH (≥6 months of age) with a child who agrees to participate in the longitudinal cohort will be invited to participate in the febrile surveillance. The HH will be invited to participate if the adult head of HH agrees to monthly census, health visits, and logging resident symptoms when febrile, if the residents spend 4 h per week in the house or sleep in the house, and the study personnel does not identify a security risk. Then each resident of that HH will be invited to participate in febrile surveillance if they are ≥6 months of age and spend a minimum of 4 h per week within the HH or sleeps in the house. Inclusion criteria will also include only those individuals who self-report a fever event or report of feverishness within the previous 24 h of presenting with a rash, arthralgia, arthritis, or non-purulent conjunctivitis, or present with fever at time of study health facility visit.

Children will be eligible for inclusion in the longitudinal cohort if they are 4 to 16 years of age, are resident of the HH or frequent visitor (spending at least 20% of their day in the residence), and plan to stay in the same residence or study area for at least 24 months.

If the head of the HH consents and the HH is eligible, the HH will be enrolled to receive the Mosquito Shield™ application and be part of the monthly entomological surveys.

### Who will take informed consent? {26a}

Consent will occur at potential participant homes following community sensitization. Informed written consent will take place for each component of the study: febrile surveillance (clinical case monitoring including HH census and participant movements along with child assent); longitudinal cohort (including future use of specimens and research information along with child assent); SR product application; and entomology surveys. Consent will be sought from adult individuals who agree to participate for febrile surveillance. Consent will be sought from parents/guardians of children to participate in the febrile surveillance cohort (≥6 months–16 years) and/or longitudinal cohort (≥4–16 years). Both a parental/legal guardian signed ICF and assent to participate will be obtained for persons ≥8–16 years of age. Consent will be sought from heads of HHs or his/her spouse for HH census, assessing resident movement, receiving intervention (including allowance of study staff to replace products every 28 days and perform periodic spot checks), and entomology surveys.

In all cases, written consent will be obtained. If the participant or guardian is not able to write, they will provide a thumb print on the consent form for documentation of willingness to participate, and a witness will sign to confirm the person has consented to participate. The study will be explained in the local languages (Sinhala and Tamil). Time will be granted to those parent(s)/guardian(s) who would wish to make consultations with their family members before signing. It will be stressed to all parents/guardians approached that their children’s entry into the study is voluntary, and they may withdraw from the study at any time for any reason without any penalty. Study staff will ensure all questions have been addressed before the consent from is signed. Consented subjects will be assigned a unique subject identity code and be screened for inclusion/exclusion criteria on enrolment.

### Additional consent provisions for collection and use of participant data and biological specimens {26b}

Consent will be obtained for future use of research specimens and/or information. Any future studies that may be performed using the blood samples collected during this study will most likely be related to dengue or other diseases transmitted by insects, for instance (1) currently uncharacterized viruses may be discovered over the course of the project and it might be advantageous to screen subject samples for such viruses, (2) it might be necessary to revalidate/reconfirm the original laboratory findings, (3) if new and improved serological/diagnostic tests are developed whilst the project is already in progress it would be beneficial to the project if we could use them to confirm the findings. In the future, some of the research may help to develop new products, such as diagnostic tests and drugs.

On completion of routine testing, all remaining samples will be kept at the Epidemiology Unit in Sri Lanka for up to 5 years from the end of the study. Any remaining samples will be destroyed. A subset of PCR-positive samples will be used for virus isolation at the University of Sri Jayewardenepura Research Laboratory, Colombo. Those virus isolates will be stored at Epidemiology Unit/National Dengue Control Unit (NDCU), where a reference collection of Sri Lankan DENVs will be housed and will be available for potential use in future DENV genetic studies (for up to 5 years) for building onto current data of DENV serotype circulation patterns and other DENV characterization laboratory-based studies.

## Interventions

### Explanation for the choice of comparators {6b}

According to the WHO VCAG’s guidelines for vector control field trial design, studies should always have a control group from which data collection occurs contemporaneously with data collection from the intervention group [36]. Our trial design includes a placebo product of matched design to the Mosquito Shield™ but with inert ingredients only. The use of a placebo is generally acceptable when a placebo is compared against an intervention in combination with standard treatment [37]. Our study design will not withhold standard-of-care for clinical management of dengue nor standard-of-care vector control interventions in the placebo arm but will be monitored and recorded throughout the trial. This approach is aligned with WHO VCAG guidance that the control group must receive care reflecting the standard best practice interventions. Study participants will not be instructed to avoid alternative vector control tools (e.g., coils, topicals, aerosol sprays, repellents). The choice to use the Mosquito Shield™ product was based on this product containing the same active ingredient (AI) and design (i.e., passive emanator) as was used in clinical trials dating back to 2013 which demonstrated impact to reduce malaria and arbovirus infections [4, 29]. Thus, preliminary public health value data exists for this “first-in-kind” prototype for the SR class.

### Intervention description {11a}

The SR will be a new formulation of transfluthrin, Mosquito Shield™. This AI is currently widely used in mosquito coils and other HH pest control products on the global consumer market. The new formulation is a passive emanator that will release AI over a period of 28 days without the need of electricity or external heat. The product will have a standard amount of AI that will be released throughout the treated space continuously based on a standardized replacement schedule. A placebo product of matched design with inert ingredients will be applied similarly. The Mosquito Shield™ and placebo products for this study will be designed and provided by S.C. Johnson, Inc., A Family Company.

If the formulated product is not registered in the study area, an experimental use permit will be obtained before the study begins. Industry partners will manufacture, package, and deliver products according to site-specific sample size estimates. A preparatory time period (ramp-up phase) will be factored into the study to facilitate product acquisition in-country, consenting HH measurement and infrastructure requirements for HH application within clusters. HH characterization will include recording number of doors, windows (status of screening) to support results interpretation of risk of transmission or infection as a function of host odor concentration in the house. Existing national standard vector control interventions (i.e., bed nets and insecticide fogging) will continue without interruption in study clusters throughout the trial, but will be monitored and recorded. Study participants will not be instructed to avoid alternative vector control tools (e.g., coils, topicals, aerosol sprays, repellents) in either study arm.

Mosquito Shield™ and placebo products will be placed and replaced inside consented homes by dedicated, trained study staff according to the manufacturer’s specifications. All HHs will receive two products for every 9 m^2^ area. More than one emanator may be applied in a HH depending on the size of the house (i.e., a HH 100 m^2^ would require 12–16 sheets). Each product will have a batch number from the industry partner(s) and a unique identifying code (UIC) associated with an individual cluster, which will be recorded at the time of installation and replacement. Industry partners will ensure that the listing of UICs (identifying the contents as having an AI or placebo) is accurate and kept strictly confidential. All consenting HHs within the intervention arm (study cluster) will be assigned active products, with all consenting houses within the control arm (randomly chosen) receiving blank/placebo. Initial product application, and later removal and replacement, will require approximately 20 min.

Intervention quality assurance will be addressed at the time of manufacturing, as well as, during the trial. As part of the manufacturing process control plan, incoming transfluthrin purity and the transfluthrin content in the formulation were analyzed for every batch by gas chromatography. SR and placebo intervention filling weights will be measured every hour during production. To verify the amount of transfluthrin in Mosquito Shield™ and the absence of transfluthrin in placebo products that had been received in Sri Lanka, an independent analysis of a subsample of unused interventions will be performed by Ross Laboratories, India.

### Criteria for discontinuing or modifying allocated interventions {11b}

If a study participant chooses to end their participation, study staff will respect the individual’s decision without penalty. If a study participant no longer meets the study’s inclusion criteria and/or based on adverse event (AE) and/or serious adverse event (SAE) clinical assessment, staff may terminate subject participation at any time during the trial.

### Strategies to improve adherence to interventions {11c}

In order to promote adherence to intervention protocols, study staff will be employed to ensure the timely replacement and accurate placement of the SR products. Product monitoring will be conducted during product replacement and entomological surveys (every 28 days). Unannounced visits to randomly selected homes in each cluster (up to 1% of homes within a single cluster) will occur monthly throughout the intervention phase of the trial by trained, study staff. This will mitigate bias due to HHs simply complying when knowledge of product replacement will occur.

Monitoring will include documentation of product presence and quantity in the house as well as verification that the UIC of products matches that assigned to the Household Identification Number (HIN). Mismatching UIC and HIN will be promptly reported to study product manager(s) for protocol management and/or staff refresher training.

A HH will be labelled “compliant” if product UIC and HIN matches and the required quantity of products according to manufacturer specifications (2 units/9 m^2^) are in the home at time of inspection. Overall product coverage will be estimated based on total HHs recorded as having correct product volume at time of replacement. If the inspector finds deviation from prescribed application, the HH will be recorded as “non-compliant” and a report detailing the nature of non-compliance will be submitted on the same day. There will be no consequence to the homeowner if a house is identified as non-compliant. If study staff observe a product has been moved after application during a scheduled product replacement, the move will be recorded for use in HH compliance assessment. If necessary, study staff will re-engage with heads of HHs on the importance of maintaining original product placement. Product compliance summary reports will be produced to summarize compliance in the distribution, schedule, and HIN/UIC concordance for use in analyses to inform product development and guide implementation strategy to ensure end-user compliance and acceptability in the future.

### Relevant concomitant care permitted or prohibited during the trial {11d}

Existing national standard vector control interventions (i.e., larviciding, source reduction, and insecticide fogging) will continue without interruption in study clusters throughout the trial but will be monitored and recorded. Study participants will not be instructed to avoid alternative vector control tools (e.g., coils, topicals, aerosol sprays, repellents, ITNs) in either study arm. These approaches will allow for an estimation of the SR effect assuming all other measures are still occurring for arbovirus prevention, essentially providing insight on an additive benefit above that provided by the application of currently recommended WHO arbovirus preventive measures.

### Provisions for post-trial care {30}

Not applicable—the study will not provide post-trial care.

### Outcomes {12}

The primary outcome measure is the incidence of ABV infection in the longitudinal cohort. The primary endpoint is the fraction of monotypic or seronegative individuals in the longitudinal cohort who seroconvert to an arbovirus during the follow-up period post randomization with intervention. The intervention follow-up period is 2 years after initial deployment of SR or placebo. There will be 3 blood samplings from longitudinal cohort participants for measure of seroconversion: one for baseline serostatus characterization (T0), a second at 12 months (T1) and a third at 24 months (T2) from time of initial placement of intervention.

#### Secondary outcome measures include:

1. Clinically apparent laboratory-confirmed cases of ABV disease in the longitudinal cohort, measured by comparing laboratory-confirmed ABV infection rates in subjects residing in HHs with active and placebo product, as an indicator for ABV disease. (Time frame: 24 months).
2. Clinically apparent ABV disease in subjects participating in the febrile surveillance cohort, measured by comparing ABV case definition rates in subjects residing in HHs with active and placebo product, as an indicator for ABV disease. (Time frame: 24 months).
3. Adult female *Aedes aegypti* indoor abundance measured by comparing adult female *Aedes aegypti* indoor abundance in HHs with active and placebo product receiving standard entomological surveillance and control procedures by the local Ministry of Health, as an indicator for reduced mosquito house entry due to effect of product. Adult female *Aedes albopictus* abundance may also be assessed. (Time frame: 24 months).
4. Adult female *Aedes aegypti* blood fed rate, measured by comparing adult female *Aedes aegypti* blood fed rate in HHs with active and placebo product receiving standard entomological surveillance and control procedures by the local Ministry of Health, as an indicator for reduced mosquito human contact due to effect of product. Adult female *Aedes albopictus* blood fed rate may also be assessed. (Time frame: 24 months).
5. Diversion of *Aedes aegypti* mosquitoes into untreated houses, measured by comparing adult female *Aedes aegypti* indoor abundance in untreated HHs adjacent to treatment clusters (with active product) to indoor abundance in untreated HHs adjacent to placebo clusters as an indicator for mosquito diversion due to effect of product. Diversion of adult female *Aedes albopictus* may also be assessed. (Time frame: 24 months).
6. Overall incidence of ABV infection, measured by the seroconversion rates of all children enrolled in the trial, independent of order of infection, i.e., including tertiary and quaternary infections. (Time frame: 24 months).

#### Tertiary outcome measures include:

1. Comparing ABV infection and disease metrics as well as entomological endpoints between participating individuals / HHs in SR clusters and individuals / HHs from the same clusters who did not agree to the SR component of the trial. (Time frame: 24 months).
2. AEs and SAEs measured by solicited and unsolicited reports from the longitudinal cohort and febrile surveillance cohort during the trial period. Mean, minimum, and maximum frequency and percentage of AEs and SAEs across clusters among enrolled subjects will be summarized by treatment arm. (Time frame: 24 months).

### Participant timeline {13}

The trial is expected to begin at the end of 2022 and continue for 2 years. Enrolment, baseline blood sampling, and intervention deployment will occur through a staggered approach, whereby clusters will be grouped by geographical distance/spatial location (Group 1, Group 2, etc.) to help manage field logistics. HHs within Group 1 will be enrolled first, followed by HHs within Group 2 and so forth. Product deployment in each cluster group is anticipated to occur in a 1–2-week timeframe. Each HH will be followed up for a total of 2 years (24 months) after the initial deployment of the product.

The schedule of activities described in the previous sections is outlined below:

1. Baseline assessments/procedures prior to intervention deployment (~3 months duration)
  Clusters delineated based on HH mapping and census data collected from the Negombo, Wattala, and Kelaniya study areas in the Gampaha District, with support of historical demographic, epidemiological, and/or entomological indices.
  House recruitment visits, distribution of information sheets, and study explanation.
  Consent and enrollment into longitudinal and/or febrile surveillance cohort(s), entomology surveys, and/or product application.
  Baseline serostatus blood sampling from longitudinal cohort participants (Baseline sample: T0).
  Indoor, adult mosquito collections using Prokopack aspirators in enrolled HHs.
  First application of intervention in consented HHs following completion of enrollment within cluster groups.

(2) Intervention phase assessments/procedures (24 months duration)
  Deployment and replacement of intervention in consented HHs (every 28 days).
  Blood sampling from longitudinal cohort participants (Annual sample T1: ~12 months from baseline sample; Annual sample T2: ~24 months from baseline sample).
  Blood sampling from longitudinal cohort participants also enrolled in febrile surveillance reporting to study health facilities at time of fever/symptoms.
  Clinical case monitoring from trial health facilities for participants in the febrile surveillance cohort; reporting at the time of fever/symptoms.
  Monthly indoor, adult mosquito collections using Prokopack aspirators.
  Disposal of intervention product.

**Table:** 

	Study period			
	Baseline	Intervention		Close-out
**Timepoint**	(2023)	(2023)	(2024)	(2024)
**Estimated duration**	Study months 1–3 **3 months**			
	Study months 1–24 **24 months**			
				Study months 25–27 **3 months**
**Baseline**				
**Recruitment, consent, enrolment**	X			
**Allocation**	X			
**Serostatus blood sampling (T0)**	X			
**Initial entomology surveys**	X			
**Initial deployment of intervention**		X		
**Intervention**				
**Intervention replacement**		X	X	
**Longitudinal blood Sampling (T1)**		X		
**Longitudinal blood sampling (T2)**			X	
**Febrile surveillance**		X	X	
**Entomology surveys**		X	X	
**Assessments**				
**Final analysis**				X

### Sample size {14}

The sample size determination for the required number of HHs per cluster for the trial is based on the risk of seroconversion comparison in a logistic regression model.

Dengue transmission is characterized by local and focal transmission. Spatio-temporally resolved estimates of year-to-year variation in transmission intensity is, therefore, difficult to predict. Recent seroprevalence studies in Sri Lanka found evidence of a relatively consistent force of primary infection of 0.141 [38]. Colombo has a higher dengue incidence rate than Gampaha (100 per 10,000 versus 77 per 10,000), so we had to adjust estimates of force of infection for Gampaha. Using reported dengue disease incidence rates for Colombo and assuming that dengue disease rates were directly proportional to secondary dengue infections, we tuned a deterministic, 4-serotype catalytic dengue transmission model to Colombo data. We then were able to identify the expected relative reduction in the force of first infection when the incidence rate was reduced by 23%. To identify the expected percent of each age group that would be wholly susceptible, we simply applied the solution to the 4-serotype catalytic model to each age. For example, ignoring maternal-derived immunity, the probability a person of age a is wholly susceptible to dengue given a yearly force of primary infection of 0.062 is (1–0.062).

Census information for the 3 MOH areas selected for the trial within Gampaha indicates a relatively constant age distribution among children and adolescents: 7.59% are 0–4, 7.80% are 5–9, and 7.39% are 10–14. Assuming that within an age bin that the age-specific distributions are uniform, we can estimate the percent of the population of Gampaha that is any age between 4 and 16 and has had zero or one DENV infections. For children between ages 4 and 16, taking population weighted averages, we estimate 58% are seronegative to DENV and 33% are monotypic.

**Fig. 2:**
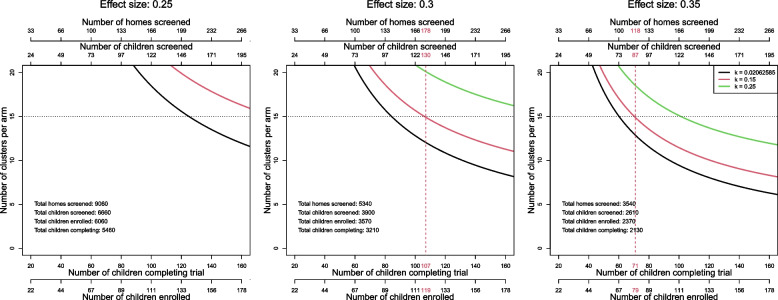
Estimated subject enrollment and cluster requirements for varied effect sizes and coefficients of variation

The average HH size in Gampaha is 3.7, of which we estimate 0.73 will be between 4 and 16 years old and wholly susceptible or monotypic. Following directly from Hayes and Moulton [39], we can calculate the minimal number of clusters required to power a trial for a given effect size and coefficient of variation. Based on previous SR trial, an effect size of 30% is conservative [4]. More informative for sample size calculations, the coefficient of variation in that trial was estimated following Hayes and Moulton to be 0.021. This was a low coefficient of variation, so for purposes of these sample size calculations, we assume the coefficient of variation is 0.15. Below (Fig. 2), we plot, by cluster, the number of children necessary to be screened and enrolled (and expected to complete the trial) and the corresponding numbers of clusters for effect sizes from 0.25 to 0.35 and coefficients of variation of 0.021, 0.15, and 0.25.

Assuming the probability of seroconversion for seronegative or monotypic individuals was 5.8% with a coefficient of variation of 0.15 and an *α* of 0.05, we estimated the need for 30 clusters (15 per arm) with approximately 110–120 qualifying individuals. This will provide 80% power to detect a minimum of 30% PE over 2 years based on a force of primary infection of 0.062 and an anticipated 80% participation rate.

Conservatively, a cohort of 14,430 participants across 3900 HHs will be enrolled for febrile surveillance of mild apparent disease. A subset of this cohort, ~3570 (110–120 per cluster) subjects will also be enrolled from the febrile surveillance cohort for measuring DENV infection. Assuming a 10% loss to follow-up (LTFU) over the 2 years of the study, this will result in 3210 subjects (100–110 per cluster) to assess the primary endpoint of DENV seroconversion.

“Qualifying participants” for the primary endpoint analyses on seroconversion are defined as individuals whose baseline serostatus to DENV is seronegative, indicating they have never been exposed to DENV, or monotypic, indicating detection of antibodies to a single DENV infection. Gampaha District is dengue endemic, and as such, our primary target group for monitoring seroconversion will be children ≥4–16 years of age who have a lower probability of having had multitypic DENV infections. Identification of seroconversions in participants experiencing 2nd, 3rd, or 4th DENV infections is difficult given the diagnostic capacity of existing laboratory assays. By restricting our qualifying population for the primary outcome measure to seronegative or monotypic individuals, we maximize the probability of generating confidently interpretable data. In addition, this group is more likely to spend much of their time at risk throughout the day in and around their home, reducing contamination effects associated with human movement. Focusing on persons ≥4–16 years of age and taking a population weighted average, we estimate 58% of potential study participants are seronegative to DENV and 33% are monotypic.

As mentioned above, a secondary endpoint will focus on all 3900 children enrolled in the trial, looking for any detectable seroconversion, i.e., including tertiary and quaternary infections. Because these are expected to be rarer and more difficult to detect, the power associated with this endpoint is estimated to be lower than the primary endpoint’s power.

### Recruitment {15}

Recruitment of participants for enrollment will be based on random selection of HHs using census mapping of the study area. Information sheets will be supplied to residents during their first visit, explaining in local dialect the study and associated study procedures. The information sheets will give information about the study, the SR product, the future use of specimens and research information, blood samples, and symptomatic cases and entomology surveys. The information sheets will be distributed through NDCU study staff with support from community interviewers and/or community health workers, as needed, targeting parent(s)/guardian(s) of potential participants in the community, community leaders, civil society members, and organizations working in the district. Information will be provided through community meetings held at the local Ministry of Health clinics where study staff will present the study and have discussions with members of the community. Additionally, study staff will attend the established local Community Advisory Board and opinion leaders meetings (i.e., women groups and men groups or equivalent) where information sheets will be read and handed to the parent(s)/guardian(s) of potential participants. Study staff will be granted opportunity to explain the study and have discussions with meeting participants.

Study staff will visit HHs to recruit and consent HHs and/or individuals into the various study components (serological sampling, entomological surveys, Mosquito Shield™ application) following HH mapping in the study area and cluster delineation. Prior to performing serological sampling, entomological surveys, or Mosquito Shield™, a census form and HH questionnaire will be administered to the head of the HH by study staff.

Information will be distributed through NDCU study staff with support from community interviewers and/or community health workers, as needed, targeting parent(s)/guardian(s) of potential participants in the community, community leaders, civil society members, and organizations working in the district.

## Assignment of interventions: allocation

### Sequence generation {16a}

The unit of randomization for the intervention and placebo will be a cluster, for a total of 30 clusters (15 per treatment arm). Criteria for stratification (as needed) will be baseline dengue incidence rates, and/or adult entomological measures (if available). Following stratification (as needed), clusters will be allocated to receive either active or placebo treatment using a random number generator (https://www.random.org).

### Concealment mechanism {16b}

The Mosquito Shield™ and placebo product, formulation, and packaging will be indistinguishable, thus investigators, study staff, and HH residents will all be blinded to treatment. Only the Data Safety Monitoring Board (DSMB) and industry partner will have access to the code that identifies the product as an active or placebo. SR and placebo intervention will be deployed in houses by study personnel using a blinded coding scheme. The study biostatistician will remain blinded throughout the trial, but will conduct an unblinded analysis following database lock upon completion of all data entry and resolution of standing data queries at the end of the study.

### Implementation {16c}

The external statistician serving on the DSMB will use a random number generator to assign clusters to SR or placebo treatment arm. All enrolled HHs within the intervention arm will be assigned active products with all houses within the control arm receiving blank/placebo. Trained study staff will enroll participants. All consented HHs will have product placed inside their homes at the manufacturer’s recommended application rate of 2 units per 9 m^2^ floor area. Trained study staff will be responsible for management of product implementation which will include the initial deployment of product, subsequent removal, and replacement at 4-week intervals.

## Assignment of interventions: Blinding

### Who will be blinded {17a}

Participants and study staff will be blinded as to whether a study cluster is receiving the active SR or placebo product. The site database manager will assign a HIN to each HH and the site intervention administrator will coordinate distribution of blinded active or placebo product to enrolled HHs in each cluster corresponding to the pre-labelled package code that aligns with cluster number.

### Procedure for unblinding if needed {17b}

The DSMB will have the product code to facilitate management of emergency unblinding on-site for the purposes of AE and SAE follow-up by a study clinician.

#### Non-emergency unblinding of a single participant

If, because of an AE which might be related to the SR product, and a non-emergency unblinding of an individual participant is being considered, unblinding will follow recommendations outlined in pre-specified standard operating procedures (SOPs) for non-emergency unblinding. The site clinician will inform the Site PI of the AE under consideration and the Site Principal Investigator (PI) will contact the UND Lead PI and the medical monitor on the DSMB to discuss the case and obtain agreement that the participant, thus HH allocation, should be unblinded in a non-emergency manner. If unblinding is agreed upon, the sealed (digital password protected) intervention assignment will be with the Site PI but only opened by a pre-designated person external to the study (i.e., administrator) so as to maintain the site PI’s and study staff blinding to cluster assignments. Documentation of the unblinding will be performed with a subsequent follow-up memo to the UND Lead PI, and DSMB. Reporting of non-emergency unblinding due to an AE will be conducted as prescribed by corresponding institutional review boards (IRB) by the Site PI, UND Lead PI, or designee. The possible effect of unblinding on the planned study data analysis will be determined by the Site PI or designee.

#### Emergency unblinding

Emergency unblinding will be considered in instances of a suspected unexpected SAE to the study product or procedures (dengue treatment, mosquito collection) as judged by a site physician following recommendations outlined in pre-specified SOPs for emergency unblinding. The first alert will be raised by a study physician within 24 h of becoming aware of the SAE in an expedited report to the Site PI, UND Lead PI, and DSMB. Documentation of the unblinding, reporting to IRBs, and possible effects of unblinding on the planned study data analysis will follow similarly as described above.

## Data collection and management

### Plans for assessment and collection of outcomes {18a}

#### Mapping of the study area and census measurements

Prior to enrolment, all structures in the study area will be mapped using GPS coordinates and assigned a unique HIN. Prior to performing serological sampling, entomological surveys, or Mosquito Shield™ application, a census form and HH questionnaire will be administered to the head of the HH. The survey will take approximately 20 min. A listing of all residents will be recorded. Each person appearing on the census will be assigned a participant code based on the HIN. This allows for trial staff to identify members of each HH if/when a member participating in the febrile surveillance cohort reports to a designated health facility. Trained trial staff will obtain the number, age, and sex of occupants, dimensions of the property, housing construction materials and design (doors, windows), method of cooking, water use patterns, type of sewage disposal, and insecticide use. The number of occupants and dimensions of the property will be used to calculate mosquito indices using persons or hectares in the denominator. The remaining information will be used to assess the requirement of pre-stratification of clusters prior to randomization to Mosquito Shield™ active or placebo treatment arms to control for confounding and account for differences in SR efficacy.

At each longitudinal cohort sampling interval (1× per year), census forms will be generated from the database and movement of individual residents into or out of enrolled HHs will be assessed. As part of the initial HH census, and 1× per month during intervention replacement, each resident will be asked to estimate the time spent at the residence during daytime hours for weekdays and weekends separately. This will allow us to control for difference in HH exposure to infected mosquito bites among participants. Each survey will take approximately 20 min to complete.

#### Screening and monitoring of cohort participants

**Fig. 3:**
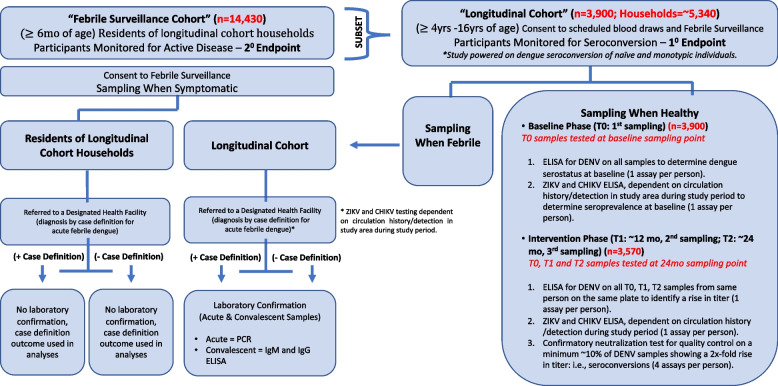
Overview of ABV transmission monitoring

A graphical representation of DENV infection and disease follow-up of study participants is outlined in Fig. 3 below.

##### Enrollment and monitoring of the febrile surveillance (clinical case) cohort

Febrile surveillance will be implemented at the initiation of the study and will continue for the duration of the study through an enhanced passive surveillance system. Every resident (≥6 months of age) of a HH with a child who agrees to participate in the longitudinal cohort (explained below) will be invited to participate in febrile surveillance and be asked to log fever symptoms. Each HH will be provided with a digital thermometer for enrolled residents to check and record temperature when feeling ill with fever (axillary temperature of > 37.5°C) or in the absence of fever, presenting with rash, arthralgia, arthritis, or non-purulent conjunctivitis in the family record book provided by study staff. During monthly house visits for intervention replacement and entomology surveys, record books will be checked and scanned.

When a participant reports fever (or in the case of suspected Zika in the absence of fever, presenting with rash, arthralgia, arthritis, or non-purulent conjunctivitis), special emphasis will be placed on the warning signs of dengue and the importance to go to a health facility first and contact us second. They will be referred to visit a designated health facility for dengue case ascertainment by clinical officers serving the health facility per routine standard of care procedures using current NDCU/Ministry of Health case definition for acute febrile dengue. Case determination outcomes (± dengue) will be reported to study staff using an assigned participant code related to a unique HIN assigned during mapping/census to ensure HH- and cluster-level association. Other health providers/practitioners in the area (not directly involved in the study) will be sensitized to the study and refer study participants with fever to a designated health facility.

For HH residents consenting to febrile surveillance, and reporting to health facilities with fever (or in the absence of fever, presenting with rash, arthralgia, arthritis or non-purulent conjunctivitis), they will be provided the results of the health officer’s dengue case ascertainment with no further confirmation testing. For participants enrolled in the longitudinal cohort, who also consented to febrile surveillance and report to health facilities with fever (or in the absence of fever, presenting with rash, arthralgia, arthritis or non-purulent conjunctivitis), they will be provided the results of the health officer’s dengue case ascertainment and, whether determined ± for dengue, requested to provide both acute (at time of reporting) and convalescent blood samples (14–21 days later) for laboratory confirmation of infection. Both acute and convalescent samples will be tested for anti-dengue antibodies by the trial laboratory. Testing of longitudinal cohort participants for ZIKV and CHIKV active infection will be dependent on circulation history/detection in study area during study period.

##### Enrollment and monitoring of the longitudinal cohort

Children aged ≥4 to 16 years of age in HHs under febrile surveillance will be screened for enrollment eligibility into the longitudinal cohort. At enrolment, the age and gender of the child will be recorded and a blood sample taken for detection of existing antibodies against DENV to determine serostatus at baseline. Follow-up serum samples will be requested from eligible qualifying participants within ± 2 weeks of when the intervention is installed (Baseline Time 0), and again ~12 (Intervention sample 1: T1) and ~24 months later (Intervention sample 2: T2), for a total of 3 sampling points over a 2-year follow-up period to coincide with two DENV transmission seasons. At 12 months, as needed, we will recruit participants who have stayed in the study area under febrile surveillance to participate in the longitudinal cohort, replacing individuals LTFU, to ensure study power is maintained.

Blood samples will be collected by venipuncture or finger prick procedures using standard aseptic techniques. An experienced phlebotomist (or study physician) will take the blood sample from an antecubital vein. For the longitudinal measuring of DENV-neutralizing antibodies, 5–10ml (1–2.5 teaspoons) of whole venous blood will be obtained from each volunteer. Blood will be collected in one Vacutainer collection tube (red top) without anticoagulant. If venipuncture is unacceptable to a study subject, the finger prick method will be used for blood collection. Providing each participant with a choice of methods will increase compliance and reduce the drop-out rate during subsequent collections. For finger pricks, we will use a BD genie safety lancet and microtainer tube system. After cleaning a finger with 70% alcohol, the sterile lancet will be used to the puncture skin. The finger will be squeezed by the phlebotomist forming a large drop of blood which is held over the microtainer tube. Capillary action draws the blood into the 1-ml tubes. Sera will be separated by centrifugation at 2500 rpm for 10 min at 40°C, transferred to cryo-vials and stored at −20°C.

If necessary, the project physician will evaluate the participant and conduct medical exams (remotely or in person) every 3 days for the course of the illness (usually 3–7 days). The medical visits will take approximately 20 min. Ill subjects will be referred to primary healthcare clinics or hospitals where treatment is free. If medical exams yield suspicion of DHF/DSS participants will be transported to a local public hospital where their treatment for dengue is free.

#### Entomological surveys

Entomological monitoring will be conducted in all enrolled HHs of longitudinal cohort participants that consent to surveys. Using indoor, adult Prokopack mosquito aspirators [40], sampling will occur 1 time prior to intervention deployment and/or at time of intervention deployment, and thereafter 1 time per month during the entire intervention period, to coincide with product replacement (every 28 days).

##### Indoor aspiration

Surveys are anticipated to require 20–30 min per HH and will be conducted by trained study staff to monitor indoor adult *Ae. aegypti* mosquito populations using Prokopack aspirators [40]. All adult *Ae. aegypti* per sampling period per cluster will be assessed for blood fed status, scored as unfed, blood fed, or gravid. Adult *Ae. albopictus* that are captured may also be assessed for blood fed status.

##### Diversionary effects

Diversionary effect (DE) defined as increased mosquito movement to untreated structures in response to sub-lethal effects of AI will be assessed using the entomological measure of adult female *Ae. aegypti* indoor abundance. Fixed, sentinel houses will form an exclusive sampling frame for DE assessment and be excluded from subject recruitment, for either seroconversion or febrile surveillance, as well as intervention application.

This pattern of recruitment will be used to ensure DE sentinel houses are located outside study cluster geographic boundaries, but within a typical *Ae. aegypti* flight distance from nearest HH(s) with intervention; which will be ~10–30m [41]. Either a standardized number of DE houses will be recruited for each study cluster, based on the structure and physical features of the clusters, or a fixed proportion of total houses in the associated study cluster determined by logistical and/or resource constraints. Each DE sentinel house will have a unique HH ID associated with a specific study cluster ID (i.e., DE1 – Cluster 1) to facilitate association with SR or placebo intervention during blinded data analyses.

Sentinel houses will be sampled 1× monthly, following the same procedures described in above, during pre-intervention and intervention study periods using indoor Prokopack aspirators by trained trial staff. Sampling during the intervention period will coincide with pre-specified intervention replacement schedule in the DE-associated cluster. Estimates of increase in adult female *Ae. aegypti* abundance in DE sentinel houses during intervention from pre-intervention study periods will be compared by treatment allocation between (1) DE houses and houses within associated cluster and (2) DE houses alone.

##### Monitoring for vector insecticide resistance

To measure the innate resistance of the local, wild population, mosquitoes will be tested in Centers for Disease Control (CDC) bottle bioassays using transfluthrin at a diagnostic dose of 0.2mg per bottle and permethrin at a diagnostic dose of 43μg per bottle. Baseline vector insecticide resistance of major vector species, identified morphologically, will be assessed using F1 progeny from immature collections prior to implementation of the intervention in study clusters. Assessments will be repeated at mid- and post-intervention period, to determine the effect of the Mosquito Shield™ intervention on inducing species-specific insecticide resistance levels across arms of the study.

Insecticide resistance levels will be classified according to CDC guidelines [42]. Bottles will be coated the day before by adding 0.2mg of transfluthrin in 1ml of acetone. The solution will be swirled around the bottle and then the cap opened to allow the acetone to evaporate. For the assay, 20–25 mosquitoes will be added to the bottles and knockdown monitored every 10 min up to 60 min. Thereafter, mosquitoes will be removed and placed in paper cups with access to sugar. Mortality will be scored after 24 h. Alternatively, resistance will be assayed using the WHO filter paper assays following standard protocols [43]. Insecticide susceptibility test papers impregnated with pyrethroid will be purchased from WHO collaborating center at the appropriate discriminating concentration.

#### Laboratory procedures

##### Baseline phase sampling (T0: 1st sampling)

All blood samples collected at baseline for the longitudinal cohort will be assayed by serotype-specific indirect enzyme-linked immunosorbent assay (iELISA) to determine dengue serostatus at baseline (4 assays per sample by iELISA). Testing for ZIKV and CHIKV will be dependent on circulation history/detection in the study area at time of sampling.

Intervention phase sampling (T1: ~12 months after initial placement of intervention, 2nd sampling; T2: ~24 months after initial placement of intervention, 3rd sampling)

All blood samples collected for the longitudinal cohort during the intervention phase of the study will be assayed by iELISA (not serotype specific) for DENV from same person on the same plate to identify a 2–4× fold rise in titer. ZIKV (iELISA) and CHIKV (IgG ELISA) screening and confirmatory testing will be dependent on circulation history /detection during study period. A confirmatory neutralization, either Plaque Reduction Neutralization Test (PRNT) or Microneutrilization Test (MNT), will be performed on DENV, ZIKV samples showing a 2–4× fold rise in titer using standard protocols to identify seroconversions between paired samples from the same individual.

For samples collected from the longitudinal cohort manifesting with febrile illness detected through passive surveillance activities, acute-phase serum samples will be tested for DENV infection by detection of viral RNA by reverse transcription polymerase chain reaction (RT-PCR). Both acute- and convalescent-phase samples will be screened for DENV IgM antibody by IgM-capture ELISA. If appropriate, acute and convalescent samples will be tested by DENV IgG ELISA. Febrile episodes will be classified as DENV infections based on the RT-PCR, IgM serology (elevated IgM antibody titers [>1:100] in the acute sample, convalescent sample, or both), or IgG antibody serology (fourfold rise in titers between acute and convalescent samples).

##### Laboratory assays

iELISA

The iELISA is performed as described in Balmaseda et al. and Fernandez and Vázquez with minor modifications [44, 45]. Briefly, 96-well polystyrene plates are coated overnight at 4°C with 10 μg/ml of human anti-DENV immunoglobulins. After washes with PBS-Tween/1%BSA, the plates are incubated for 1h at 37°C with a mixture of the four DENV antigens diluted 1:80. Serum specimens, serially diluted in PBS-T/0.4% BSA (1:20–1:20, 480), a negative control (1:20) and positive controls (1:5,120) are incubated for 1 h at 37°C, in duplicate. After additional washes with PBS-T, Horseradish Peroxidase-conjugated human anti-DENV antibody diluted 1/6000 in PBS with 2.5% Normal Human Serum is added and incubated for 1h at 37°C, followed by washes and the addition of the substrate tetra-methyl-bencidine. The reaction is stopped at 10 min with 12.5% sulfuric acid, and the OD read at 450nm. The titer of each sample is calculated as the last dilution for which the percent of inhibition (% I) is equal to or >50. The % I is calculated as: %I = [1 − (Absorbance of the sample/Average absorbance of the negative controls)] × 100. A 4-fold increase in titer in paired year/year samples indicates a new infection. As a reference, antibody titers determined by IE are approximately one dilution higher than hemagglutination inhibition titers, as noted previously [46].

IgG ELISA

Dengue-specific IgG titers will be determined by an IgG ELISA adapted from Ansari et al. [47]. Plates (96-well format) are coated with DENV antigen (cocktail of serotypes 1, 2, 3, and 4) produced from infected Vero cell culture lysates or uninfected cell lysates as controls. Aliquots of diluted participant’s serum samples (1:100) are added to two dengue antigen-coated wells and to two control wells. After addition of horseradish peroxidase (HRP)-conjugated mouse anti-human IgG, OD values are recorded at 410 nm. Adjusted OD values are calculated by subtracting the OD of the uninfected antigen-coated well from that of the corresponding viral antigen-coated well. The cut-off OD value for determining antibody positivity will be calculated as the mean adjusted OD plus 3 standard deviations of antibody-negative control sera.

IgM-Capture ELISA

Dengue-specific IgM antibody titers will be determined by an IgM-capture ELISA adapted from published protocols [48]. Briefly, plates (96-well format) are coated with anti-human IgM antibody to capture participant IgM antibody. Virus-specific IgM are detected by the addition of dengue viral antigen, followed by virus-specific hyperimmune ascitic fluid (HMAF) and HRP-labelled anti-mouse IgG. Following the addition of colorimetric substrate, plates are read at 410 nm. All acute- and convalescent-phase samples are initially screened at a 1:100 dilution. Samples exceeding the reference cut-off value, calculated as the mean of seven antibody-negative samples plus three standard deviations, are considered IgM antibody-positive. Positive samples will be subsequently re-tested at fourfold serial dilutions to determine endpoint antibody titers.

MNT

A validated protocol adapted from Vorndam and Beltran, in which 96-well plates (TC-treated) are inoculated with Vero cells at 2 × 105 cells/mL and then incubated at 37°C, with 5% 346 CO_2_ for 2 days or until the cell monolayer was confluent [49]. Serum samples are inactivated at 56°C for 30 min, then serially diluted in triplicate in a twofold series from 1:20 to 1:1280 on a 96-well plate, along with negative serum and positive HMAF controls. Diluted virus (dilution factor determined by validation assays) is mixed with inactivated sera and incubated at 4°C overnight. A Vero cell suspension at 2×10^5^cells/mL in 10% fetal bovine serum (FBS) Eagle’s Minimum Essential Medium (EMEM) is added to each well with the serum-virus mixture and incubated at 37°C with 5% CO_2_ for 5 days. After the 5 days, the cell culture supernatant is discarded and the cells are fixed with ethanol/methanol, washed with phosphate-buffered saline (PBS), blocked with skim milk, then anti-DENV HMAF added and incubated for 2 h at 37°C, washed with PBS again, and then incubated at room temperature for 1 h with 2,2′-azinobis (3-ethylbenzthiazoline-6-sulfonic acid) substrate. Plates are read using ELISA reader (Microplate Reader359 Biotek Instruments Inc.) at 405 nm with a 630-nm reference filter.

PRNT

A modified protocol of Morens et al. will be followed [50]. Test sera will be diluted twofold in media (EMEM + Pen./Strep.) from 1:40 to 1:640. Two hundred microliters media containing 40 to 80 PFU of assay virus will be mixed with 200 μl diluted test serum and then incubated at 40 C for 15 h. In triplicate, 100 μl virus-serum mixture will be added to 0.5 ml media containing 1.5 × 105 BHK_21_ cells and then added to a well of a 24-well tissue culture plate and incubated at 370 °C with 5% CO_2_ for 3 h. The cells will then be overlaid with 0.5 ml of overlay media (0.6% Carboxymethyl Cellulose, MEM w/o Phenol Red, 10% FBS, 0.075% NaHCO3 and Penicillin/Streptomycin) and incubated at 370 °C with 5% CO_2_ for 5 days. The media will be removed, and the cells rinsed with H_2_O and stained with 0.5 ml/well stain solution (0.1% (w/v) Naphthol Blue Black, 1.36% (w/v) Sodium Acetate, and 6% (v/v) Glacial Acetic Acid) for 30 min. The stain will be removed and the plaques will be counted. The results will be expressed as the serum dilution, determined by probit analysis that reduced the number of plaques by 70% compared to that of normal human serum at the same dilution.

RT-PCR

Viral RNA will be prepared from 140 μl of each sera sample using QIAamp Viral RNA Mini Kits following the manufacturer’s instructions (Qiagen Inc., Valencia, CA 91355). Nested DENV RT-PCR will be performed following the protocol of Lanciotti et al. on serum samples for dengue viral RNA detection [51].

### Plans to promote participant retention and complete follow-up {18b}

Participant retention strategies will include fostering relationships with participants as they engage in ancillary care at study clinics, provision of Clinical Officer’s contact information for ease of communication, participant tracing when they miss appointments, visit reminders, request participants to inform study staff of moves outside or within the study area, and periodic generation of retention rate to evaluate strategies. A study SOP will be developed to provide more details on retention activities to be conducted by trial staff.

### Data management {19}

A combination of standardized paper-based or digital forms (under Android tablets) will be used for data collection.

NDCU and UND/Center for Research Computing (CRC) will work together to develop the quantitative forms to be uploaded on tablets. All data issuing from the electronic data collection system will follow the same data collection processes outlined in the paragraph below. Any changes to quantitative data forms will need to be agreed by the site PI (in consultation with external consultants), UND PI, and Scientific Director. In the event that these individuals cannot find a consensus on proposed changes, UND’s PI will make the final decision.

The legal entity responsible for research samples will be NDCU.

#### Data sharing policy

Any data collected on paper forms (including consent/assent forms) will be scanned and transferred to binders for storage in a secure and locked restricted access area, while all electronic captured data will be archived with a documented history of changes or corrections at the local study site. Using CommCare, NDCU will collect data, which will be securely stored on Android devices and then synchronized, to CommCare cloud. Data will then undergo an initial cleaning and de-identification by NDCU, before being synced to UND’s central database. Data from NDCU to UND will be transferred through a dedicated secure sFTP server with password-protect access from NDCU.

The trial will generate considerable data and biological samples over the course of the proposed study period. The data management plan will follow the guidelines and suggestions put forward by the NIH in its online guidance document: https://osp.od.nih.gov/scientific-sharing/nih-data-management-and-sharing-activities-related-to-public-access-and-open-science/ [52] and by the respective institutions involved in the research, as well as the community of interest (comprised of colleagues, scientists working in the same field, the biomedical community researching tropical parasitic diseases, and public health officials). The goal is transparent sharing of key findings and data so that the broad impacts of the research are meaningful and useful to key stakeholders and will, therefore, be shared with stakeholders as may be required.

Every consideration will be given to the nature of beneficence and justice expressed in the guiding documents for research on human subjects. Biological samples from mosquitoes and viruses will be maintained appropriately to avoid deterioration. These materials will be made available to researchers upon reasonable request and with the caveat that any forthcoming publications from research on the samples should consider the original researchers and their inclusion in the publications should the situation warrant it.

De-identified data may be reviewed by the NDCU, Ministry of Health, Sri Lanka and the UND IRBs and the WHO VCAG for public health value assessment of the SR product class.

#### Data storage

Standardized data forms using paper or android tablets will be used across sites such that variable codes can be cross-referenced during final analyses. Any data collected on paper forms will be scanned and transferred to binders for storage, kept under lock and key in steel cupboards provided for the trial in a suitable building identified by the Epidemiology Unit and NDCU in Sri Lanka. Maintaining this level of security will be the responsibility of study investigators at the Ministry of Health, Sri Lanka. A Clinical Monitor from an independent clinical monitoring organization, fhiClinical, will conduct periodic monitoring visits to ensure the data is stored in a secure and safe manner and only authorized study staff will have access to the data. After the study is completed, data will be kept for up to 5 years at the Epidemiology Unit in a locked and secure room. All electronic captured data will be archived with a documented history of changes or corrections at the local study site.

All samples are to be collected, stored, and analyzed in Sri Lanka. Blood samples will be sent to University of Sri Jayewardenepura Research Laboratory with a request form giving basic identifiers. Results of laboratory testing (primary and specialist diagnostic tests) will be entered into a computerized database and transferred to project office as the test results become available. Final computerized data will be sent to Epidemiology Unit monthly for analysis and preparation of summary reports. On completion of routine testing, all remaining samples will be kept at the Epidemiology Unit for up to 5 years from the end of the study. Any remaining samples will be destroyed.

A password-protected central study database warehousing data will be developed and managed by the UND and serve as a data repository and utilized for safe data storage, extraction, integration, and analysis. The data warehouse and file repository will be backed up weekly at the local server level to ease recovery as needed.

In addition, data will be stored and backed up on CommCare cloud. Access to study data is controlled through centralized administration and access will be granted to select study personnel determined by the study PI. Research records for all study subjects including history and physical findings, and results of consultations are to be maintained by the local site PI in a secure storage facility and by UND and NDCU, for up to 5 years after the end of the project or until notified by grantee. If a subject voluntarily withdraws from the study prior to the end of the trial, research records for that participant will be stored under the conditions specified for the project. Data and samples will be destroyed at any given time in those 5 years. Destruction will be verified through formal notice by the local PI.

A subset of PCR-positive samples will be used for virus isolation at the University of Sri Jayewardenepura Research Laboratory, Colombo. Those virus isolates will be stored at Epidemiology Unit/National Dengue Control Unit, where a reference collection of Sri Lankan DENV will be housed and will be available for potential use in future DENV genetic studies (for up to 5 years) for building onto current data of DENV serotype circulation patterns and other DENV characterization laboratory-based studies.

#### Data quality control and quality assurance

Data management trial staff will be responsible for verifying data accuracy and assuring data collection is following standardized protocols. These activities will promote data quality and ensure the trial is performed in compliance with Good Clinical Practice (GCP) and the applicable regulatory requirements(s). Training on data collection will occur prior to the start of subject recruitment and throughout the trial period with refresher trainings. Standardized data collection forms will be used and source data verification will occur through three primary mechanisms: (1) self-quality checks, making sure data forms are fully completed; (2) data queries, quality checks on a routine basis; (3) external monitoring, by fhiClinical, the clinical research organization responsible for trial oversight. In addition, tablet-based digital forms which will be used for data entry will be custom designed to include rules and conditions for data variable responses, e.g., text responses cannot occur for numeric value and thresholds for numeric data.

### Confidentiality {27}

Participant privacy/confidentiality protection will be assessed during routine quality assurance activities and based on the SOPs developed for the project. A Privacy Impact Assessment will be developed for the project, and a set of protocols and contingency plans for emergency paper-based and digital data destruction will be developed in order to guarantee privacy of research subjects in case of unforeseen risks.

All participant information will be confidential. Subject names will be captured on consent forms for assurances of informed consent and to assign sample codes accurately. Subject HH location data is needed for clinical follow-up, as needed. Sensitive information related to trial will be held confidential. Research documents (to include all entomological, serological, and clinical data) will be stored on secure data servers and kept strictly confidential. Any necessary paper copies of research documents will be stored at the study’s management office (NDCU, Ministry of Health, Sri Lanka) and at the Department of Immunology and Molecular Medicine, Faculty of Medical Sciences, University of Sri Jayewardenepura. Scientists at those laboratories will keep them private.

Each HH and subject will be given a unique identification code so that data generated from a HH or a subject will be linked to these codes, and homeowner or subject names will not be used. The code will be kept by the UND CRC and securely at the NDCU site according to site-specific IRB specifications and requirements for emergency situations. Outside of our database these codes will not be interpretable, rendering the data effectively unidentifiable without access to our servers. In case of an AE or SAE, confidential information may need to be shared with a study health officer in order to find a HH to follow up with proper clinical management.

The results of this study will be made publicly available to sponsors of the trial, but personal information will not be provided to anyone. If information from the study is published or presented at scientific meetings, participant names and other personally identifying information will not be used.

### Plans for collection, laboratory evaluation, and storage of biological specimens for genetic or molecular analysis in this trial/future use {33}

This study does not include genetic or molecular analyses. Standardized protocols will be developed for the collection, storage, use, and post-trial destruction of blood samples collected as part of the trial.

## Statistical methods

### Statistical methods for primary and secondary outcomes {20a}

Data analyses associated with the longitudinal and febrile surveillance components of the study will identify quantitative relationships between dengue incidence (overall, asymptomatic, and symptomatic) and entomological parameters (mosquito population densities and blood feeding status).

### Incidence calculations and case classification

In this section, we define (1) predictive and response variables for pattern and regression analyses and (2) the sources of data from the longitudinal study (longitudinal blood samples) and active surveillance. After each longitudinal sample, all participants in the project will be placed into one of the following categories: LTFU, not DENV infected, inapparent infection, symptomatic, or symptomatic with hemorrhagic manifestations (Table 5). Serologic data on people who are LTFU will be excluded from incidence calculations for the sample period prior to leaving the project.

**Table 5:** Case classifications for longitudinal samples

Case classification	Description
LTFU	Participants who move from the study area or choose to stop participation in febrile surveillance.
Not DENV infected	No change in PRNT or MNT results between longitudinal bleeds
Inapparent infection	Change in PRNT or MNT results consistent with a seroconversion between longitudinal bleeds without indication of febrile illness coincident with a laboratory-diagnosed DENV infection. Seroconversion meaning Baseline=Negative or Monotypic, 12- and 24-month sample=positive for dengue antibody or positive for antibody against a new DENV serotype in the case of monotypics.
Symptomatic infection	Clinical case identified in passive febrile surveillance with laboratory confirmation of DENV infections (see Fig. 3), which may include laboratory-confirmed seroconversion from acute and convalescent blood samples.

Symptomatic infections identified in the passive surveillance cohort will be used to calculate disease incidence, i.e., clinically apparent cases divided by the number of person-days of weekly surveillance. Using a person-time approach, we can adjust for HHs that did not make health facility visit(s) or when people leave the area for work or vacation and return later. Seroconversion rates will be calculated from those participating in the longitudinal cohort by identifying individuals whose serostatus changes between blood draws. The asymptomatic to symptomatic case rate will be calculated from this group of participants. Illness and seroconversions will be linked to the home residence. We recognize that participants can be infected away from their homes.

### Entomological analyses

With the information generated from entomological surveys, we will calculate the following adult indices of *Ae. aegypti* density to compare between SR and placebo treatment arms: adult index (AI = % houses infested with adult *Ae. aegypti*), adults per household (AA/Hse = No. adult *Ae. aegypti* collected/No. households surveyed), adults per person (AA/per = No. adult *Ae. aegypti* collected/No. people living in households surveyed), adults per hectare (AA/Ha = No. adult *Ae. aegypti* collected/No. hectares surveyed [sum of lots surveys]), adult female index (AFI = % houses infested with adult female *Ae. aegypti*), adult females per household (AAF/Hse = No. adult *Ae. aegypti* females collected/No. households surveyed), adult females per person (AAF/per = No. adult *Ae. aegypti* females collected/No. people living in households surveyed), and adult females per hectare (AAF/Ha = No. adult *Ae. aegypti* females collected/No. hectares surveyed).

### Efficacy evaluations

Seroconversion rates between SR and placebo areas can be compared directly by chi-square analysis, but more complex multivariate logistic regression models will be developed between predictive variables (mosquito indices, Mosquito Shield™ coverage, treatment area) and response variables (seroconversions, symptomatic disease). Additional analyses with epidemiological data will include comparing cumulative infection incidence (number of events divided by the total person-time at risk) in the cohort with Mosquito Shield™ to the cohort with placebo. Analysis will be carried out at multiple geographic scales starting at the individual household to block to zone/Grama Niladhari division level. PE will be measured based on number of dengue infections powered for statistical significance. Analyses will be conducted to compare PE on DENV infections between control and treatment arms using the following equation: Equation 1: PE = = [1−(Ic/I0)] × 100%, where I0 = the number of new infections in placebo houses and Ic = the number of new infections in active households (www.OpenEpi.com).

To identify differences in mean abundance of numbers of adult *Ae. aegypti* mosquitoes (broken into physiological status groups = unfed or blood fed), we will use negative binomial regression model procedure (PROC GENMOD) of SAS [53]. Models will be constructed for the dependent variables (i.e., AD/HA), and treatment area (Mosquito Shield™ or Control) will be the primary independent variable. Possible confounders such as lot size, human population density, block coverage rates, or other HH characteristics will be included if potential confounding is observed. Least square means will be used to test differences among mean rates within main effects and interactions terms, when appropriate main effect variables were then classified into groups and differences were tested using contrast statements in PROC GLM [53].

### Role of human movement

Because *Ae. aegypti* is a daytime biting mosquito, it is expected that the daytime activity patterns of human hosts will profoundly affect their risk of exposure to infective mosquitoes. People’s movement patterns, despite being hard to measure, clearly contribute to the geographic scale at which ABV transmission occurs impacting our ability to evaluate an interventions efficacy, as well as determine an individual’s specific exposure to DENV transmission. Thus, there is a need to quantify time spent in and outside the area where an intervention has been deployed (the home).

A categorical variable, representing low, medium, and high amounts of time spent away from home will be created. Time spent away from home per week will be summarized into a bivariate variable where “high movers” represent the tercile that spends most time away from home. The “high mover” variable will then be added to logistic regression models estimating the association between living in an intervention HH and dengue incidence. A logistic regression analysis will be conducted to examine the association between the treatment HHs and dengue incidence among individuals in those HHs, adjusting for the extent of movement of the different individuals. The model can be simplistically represented by:

$$Dengue\ i ncidence=\beta 0+\beta 1(treatment)h+\beta 2\left( extent\ of\ movement\right)i+\left[\dots \right]+\mu i+\mu h\ \left( where\ i= individual\ level\ and\ h= household\ level\right)$$

Incidence of dengue disease and the symptomatic to asymptomatic case ratio will be calculated between the intervention and control clusters.

### Hypothesis

#### Primary hypothesis

*H0*: SR does not reduce the probability of individuals seroconverting to ABV compared to placebo.

*H1*: SR reduces the probability of individuals seroconverting to ABV compared to placebo [seroconversion odds ratio (OR) between SR and placebo is <1; expected OR is 70% or PE is 30%].

#### Secondary hypothesis

##### Estimation:

1. The rate ratio of SR versus placebo on ABV disease will be estimated.
2. The change from baseline to post-deployment in average HH indoor female *Ae. aegypti* abundance and blood engorged rate in SR compared to placebo will be quantified.

#### Population for analysis

The intention to treat (ITT) analysis is the primary analysis approach for both the primary and secondary objectives. The ITT population includes the monotypic or seronegative individuals within each recruited HH that received at least one SR product or placebo product per the cluster randomization schedule.

The per-protocol (PP) analysis is included as a supplementary analysis for the primary and secondary objectives. The PP population includes the subjects from the ITT population that are treated following the specifications of the study protocol without major protocol deviations. Here, we define “without major protocol deviations” as individuals who had a SR product for at least 80% of the time they participated in the study. A second PP-like supplementary analysis for the primary and secondary objectives will attempt to estimate fractional impacts of SR for individuals who only received SR products for a fraction of the follow-up period.

### Subjects who moved to a new house during the intervention follow-up period

- For a subject who moved to a different house within the same cluster, that subject will be included in both the ITT and PP analyses.
- For a subject who moved to a different house in a different cluster, the subject will be included in the ITT analysis with the original treatment assignment though the new cluster although the subject moved to might have a different intervention from the original assignment. The subject will also be included in the PP analysis if the new cluster had the same intervention as the original assignment.

### Subjects who were hospitalized for serious complicated illness (e.g., chronic illness), died, dropped out, or missed scheduled visits due to reasons not related to the ABV disease outcome or intervention during the follow-up period

For subjects that fall under this category, the available data from the subjects (up to the time point when the subjects were hospitalized, died, or dropped out; data from the scheduled visits that the subjects did not miss) will be included in both the ITT and PP analyses because the missing or absent data can be ignored (see Section 6.4 of the SAP for more details).

### Subjects who did not receive (complete) intervention due to travelling outside, mis-application, or partial application of the product

For the ITT analyses, these subjects will be included as is. For the PP analysis, these individuals will be dropped because they were not treated following the specifications of the study protocol. For the second, PP-like analysis, “travel outside” (Y or N; an individual-level covariate) and the product application rate in each HH (expected to be close to 100%) will be included as covariates if the data are not overly imbalanced between the Y and N categories for “travel outside,” and there is practically/clinically meaningful variation in the product application rate across HHs and clusters. An attempt to integrate the seasonality of arbovirus transmission with the period of time that these individuals did or did not receive the product application will be made as possible.

### Replacement subjects

Replacement subjects are defined as subjects who were recruited into the study at a time point after the intervention began to replace initially recruited LTFU subjects to maintain minimum cohort numbers. Per this definition, subjects who were absent for an extensive period of time (> 3 scheduled visits) and then returned to study to the same HH as before, or to a different HH in the same or a different cluster are not replacement subjects. As detailed tracking of individual’s movements will be conducted, these individuals will be included in secondary analyses.

If the replacement occurs in the baseline period or before the first scheduled visit of the subjects who they replace in either year of the follow-up period, then the data from the replacement subjects will be included in the primary analysis for that year / years. Data from replacement subjects will not be included in the primary analysis for PE if the replacement of the original subject (from the same cluster) occurred after the first scheduled visit of the original subject for that year. However, a supplementary analysis will be performed that includes the replacement subjects.

#### Primary endpoint (ITT population)

The primary hypothesis on PE against ABV seroconversion will be tested using a survival analysis with a proportional hazard model with an exponential distribution assumption for the baseline hazard. In particular, if *h*(*t*_*ij*_| *x*_*ij*_) is the hazard rate of the *j*^*th*^ individual in the *i*^*th*^ cluster with covariate values of *x*_*ij*_, then this individual’s hazard rate of an arbovirus infection can be written as:

$$h\left({x}_{ij}\right)={h}_0\left({t}_{ij}\right)\cdot expexp\ \left({\beta}^T{x}_{ij}+{W}_i\right)$$

where ${W}_i\sim N\left(0,{\sigma}_c^2\right)$ is the random effect of the *i*^*th*^ cluster. Covariates included are age, sex, and treatment status (SR or placebo).

If the data are extremely unbalanced in a categorical covariate (e.g., 99% HHs had the same type of walls) or if a non-ignorable portion of the subjects have missing values on a covariate (due to MAR or MCAR), that covariate may be excluded in the model.

The primary PE will be estimated as $PE=\left(1- expexp\ \hat{\beta}\ \right)\times 100\%$, where $\hat{\beta}$ is the estimated regression coefficient for the intervention group and $expexp\ \hat{\beta}$ is the estimated hazard ratio between SR and placebo. The null hypothesis of *PE* = 0% is equivalent to *β* = 0, which is tested by Wald’s test, $z=\hat{\beta}/s$ where *s* is the estimated standard error of $\hat{\beta}$, at the 1-sided significance level of 5%.

#### Secondary endpoints (ITT population)

##### PE of SR protection again incidence of ABV disease

The second endpoint on PE of SR protection against the incidence of arbovirus disease will be estimated by conducting a second survival analysis on individuals who received a SR product for at least 80% of the duration of their enrollment in the study.

##### Entomological effects of SR (on female *Ae. aegypti*)

Entomological effects will be tested using the appropriate corresponding mixed effect regression with random effects by cluster and house. For each indicator, we will use a difference in difference model, comparing the changes in each value from baseline to those measured during the trial between the treatment and control areas.

We expect substantial heterogeneity in all entomological endpoints, and as such expect to find extremely wide uncertainty intervals for estimated effects. To account for this heterogeneity in space and time, we will conduct a secondary analysis using a spatio-temporal model [54]. The model’s base structure is still either a negative binomial regression or a logistic regression, but it uses spatial and temporal splines to capture natural underlying variation in mosquito population dynamics.

### Assessment of diversion

To assess the potential for diversion of mosquitoes to houses neighboring those with the SR product, we will enroll 5–10% of neighboring homes adjacent to each cluster for monthly mosquito abundance measurements. This will begin before the trial begins to collect baseline data and then a difference in difference analysis will measure the change (or lack thereof) in mosquito abundance in houses that neighbor treatment (or control) clusters.

### Interim analyses {21b}

Not applicable—no formal interim analysis will be conducted for this study.

### Methods for additional analyses (e.g., subgroup analyses) {20b}

The primary and secondary analyses will also be carried out with the PP population, with some modification on the covariate list in the corresponding models for the seroconversion, incidence of disease episodes, and entomological endpoints. For the second PP-like analysis, “travel outside” (Y or N; an individual-level covariate) and the product application rate in each HH (again, we define “greater than or equal to 80% of participation time” as the PP threshold) will be included as covariates if the data are balanced between the Y and N categories for “travel outside” and there is practically/clinically meaningful variation in the product application rate across HHs and clusters.

As possible, individuals within SR clusters who either do not consent to the SR component of the trial or who enter or leave the trial during the follow-up period may provide an opportunity to assess possible DEs of the SR intervention. Individuals within SR clusters who do not receive the SR product may still consent to the entomological collections, the active febrile surveillance, or, as applicable, the yearly blood draws for ABV seroconversion. A priori, there is no guarantee that a large fraction of individuals in SR clusters will agree to participate in the secondary data collection but not the actual SR intervention, and thus it is unclear if there will be power to detect any evidence of DEs (or the lack thereof). That being said, comparisons similar to those described above on both ABV endpoints and entomological endpoints between those who agree to the SR intervention and their neighbors who do not agree will be conducted. AEs and SAEs will be tabulated and documented.

### Methods in analysis to handle protocol non-adherence and any statistical methods to handle missing data {20c}

Per protocol, the longitudinal cohort subjects are checked for their ABV serostatus (the assay outcome) yearly. If a subject missed one or more scheduled visits, the subject will have missing values on the outcome that can be regarded as ignorable missingness. If a subject drops out study due to reasons unrelated to the SR product and/or ABV infection, then the missing observations from the subject can be regarded as ignorable missingness. In both cases, all available data from the subject will be included in the primary and secondary analysis, without employing any specific technique to deal with the data. If a non-ignorable portion of the subjects have missing values on a covariate (due to missing at random or missing completely at random), that covariate may be excluded in the model.

### Plans to give access to the full protocol, participant-level data, and statistical code {31c}

The statistical analysis plan and analytic code will be made open access. The data and supporting information will be made available 12 months following completion of data analysis and will remain open access in the public domain.

## Oversight and monitoring

### Composition of the coordinating center and trial steering committee {5d}

UND is the study sponsor responsible for overall program management and reporting. The coordinating personnel at UND will include the Lead PI, Scientific Director, Program Manager, Program Coordinator, and Finance Manager. The NDCU, Ministry of Health, Sri Lanka field teams are responsible for in-country study implementation and local oversight. They will work hand in hand to conduct this cRCT in Gampaha District and facilitate assurances of cultural and social customs, practices, and taboos in country. Representatives from UND, NDCU, and RemediumOne will support the data management team to oversee the development and implementation of data collection, recording, and cleaning. The University of Washington is responsible for statistical analysis. Clinical trial oversight and monitoring of study processes will be provided by fhiClinical, which includes but is not limited to checking enrolment, GCP training for study staff, ensuring subjects are properly consented, data quality, safety events are documented and reported as required, investigational product is managed and distributed per specifications, and study close-out activities occur on a timely basis. The DSMB will provide data and safety monitoring. SC Johnson, A Family Company (SCJ) will oversee product registration, manufacturing, packaging, and shipping to the study site.

### Composition of the data monitoring committee, its role and reporting structure {21a}

The DSMB reviews safety data about the Sri Lanka cRCT on an ongoing basis in order to monitor and rapidly identify any accumulating safety issues from across the program and may provide recommendations about stopping the study for safety reasons. Additionally, the DSMB provides additional credibility to study quality, by reviewing summary reports from PIs during baseline and intervention phases and making recommendations as needed about adjustments for study quality reasons. The DSMB consists of a Chair, Medical Monitor, DSMB statistician, and independent statistician.

Safety data will be reviewed routinely and regularly by the DSMB Medical Monitor. If significant concern is raised, he/she will engage with the committee. Summary of AEs/SAEs and death reports observed during the studies will be reviewed by the entire committee at pre-determined checks (quarterly). This will include comparison of the rate of AE/SAE in the two study arms and at the individual study site. The DSMB will be notified of any SAEs that are “at least possibly related” to the research product as they are reported to PIs. Unblinded efficacy data will be analyzed according to a pre-defined SAP by the Study Statistician at the end of intervention follow-up with outputs verification conducted by the DSMB External Statistician. The role of the DSMB External Statistician will also be to review and interpret safety and outcome information with the other DSMB members, perhaps to request further analysis, for example. The DSMB External Statistician will contribute input to program PIs as to what subset of the SAP is to be presented at different meetings.

All members of the DSMB currently have no financial relationship to the sponsor and will not be involved in the trial conduct in any role other than that of a DSMB member. Prospective members will be asked to disclose their financial relationships with any of the sponsors and/or their competitors. The DSMB reports to the Sponsor, UND. The DSMB charter can be made available upon request to UND.

### Adverse event reporting and harms {22}

The SR product contains transfluthrin, a chemical used in currently available HH mosquito control products such as mosquito coils. Exposure to the product may cause mild eye and skin irritation; however, these effects are typically transient and disappear after time. The product may be harmful if chewed on or swallowed, so HH owners will be advised to keep it away from children. The SR product will be fixed at a position out of the reach of children and will be monitored at replacement to ensure it has not been moved. If a product is found to have been removed from its position in the HH, study staff will discuss with the HH owner to determine why it was removed and if there was any problem that led to its removal. Study staff will also reiterate safety precautions that should be taken in regard to the SR product.

An AE includes any noxious, pathological, or unintended change in anatomical, physiological, or metabolic functions as indicated by physical signs, symptoms, and/or laboratory detected changes occurring in any phase of the clinical study whether associated with the study intervention or placebo. This definition includes an exacerbation of pre-existing conditions or events, intercurrent illnesses. A SAE is any untoward medical occurrence that results in death, is life threatening, results in persistent or significant disability/incapacity, requires in-patient hospitalization or prolongation of existing hospitalization, or is a congenital anomaly/birth defect in the offspring of a study subject. In addition, important medical events that may jeopardize the participant or may require intervention to prevent one of the other outcomes listed above will be considered serious.

AEs to be recorded as endpoints will be pre-defined based on SCJ. Toxicology reports of “probable,” “possible,” “plausible,” and “unlikely.” “Probable” includes sensory irritation (oral and dermal). “Possible” includes nausea/vomiting (oral), skin irritation/rash (dermal), and runny nose (inhalation). “Plausible” includes salivation, and “unlikely” includes eye irritation and headache. AEs will be documented in terms of a medical diagnosis. When it is not possible to make a specific medical diagnosis, the AE will be documented in terms of signs and/or symptoms observed by the investigator or reported by the subject at each study visit. Any hospitalization will be considered a SAE. Deaths detected in the study clusters will be recorded and reported as SAEs.

To assess AEs and SAEs associated with study procedures, to include intervention exposure, we will report both solicited (detected during febrile surveillance, monthly entomology surveys, product replacement, and/or review of family log books) and unsolicited (calls to study team). To better assess mild or unreported reactions to the intervention, we will also record the reason provided by families that may withdraw from the intervention component of the study.

All participants will have telephone contact information for the site PI and study clinician, which will be included on each information sheet and ICF. Residents will be instructed to call these numbers to report safety concerns and asked to report the safety concern. Anyone experiencing AEs or SAEs will be referred to seek care at study clinics. A digital data form will be developed for study staff to report AEs and SAEs in a standardized format. A study physician will evaluate AE and SAE complaints within 24 h of notification and provide a detailed report to the site PI who will manage further reporting according to local ethical assurance approvals.

SAEs will be reported to the local IRB, the Sponsor, and the study DSMB within 24 h of the site PI reporting the SAE. The initial alert will be an automatic acknowledgement in the database system, which will be followed within 7 days by a detailed clinical description of the SAE. All AEs and SAEs of study participants will be reported to the same groups on a quarterly basis, or according to local IRB processes. The DSMB will review safety data and can make recommendations about stopping the study for safety reasons.

AEs and SAEs will be reported in trial publications. Harms will be coded in accordance with MedDRA at time of safety outcome reporting. Summaries of symptom-based AEs, SAEs, and death reports observed during the studies will be reviewed by the trial DSMB at predetermined checks (quarterly). The AE/SAE will be labelled “Probable,” “Possible,” “Plausible,” or “Unlikely” due to SR. Summary statistics of AEs/SAEs, including mean, minimum, and maximum frequencies and percentages across clusters among enrolled subjects, will be provided by treatment arm. Statistical comparisons of the AE/SAE rates between the two arms will be conducted upon the completion of the study. Two sets of statistical analysis will be run. One set will compare the proportion of having at least one occurrence in each symptom-based AE/SAE during the whole study between the two arms, and the other will compare the total number of occurrences for each AE/SAE between the two study arms. If the data collected permits meaningful statistical hypothesis testing, *p*-values from the treatment comparisons will be reported, with multiplicity correction via the false discovery rate (FDR) approach [55].

### Risk to study participants

Risks to study participants are minimal. The only invasive procedure used to collect biological specimens from human participants is venipuncture (blood/serological sample). As such the *immediate risks* are occasional bruising and a slight risk of infection at the site of the venipuncture are the most serious injuries that a volunteer can receive from participating in this part of the study. There are no anticipated *longer-term risks* for biological sampling. Venipuncture and/or finger prick will be performed by qualified phlebotomists from NDCU. Universal standard safety precautions including using aseptic technique and single-use sterile needles will be followed when handling blood samples.

If a participant is injured as a direct result of taking part in this trial, the participant will be given medical care for that injury, at no cost to the participant. The participant will not receive any injury compensation, only medical care.

There is no expected greater risk for HHs and their occupants participating in this study due to the Mosquito Shield™. Adverse effects from the product will be mitigated following the manufacturer’s specifications and trained personnel will provide oversight for Mosquito Shield™ application. The AI is approved for human use by WHO and used in numerous consumer products throughout the world [56]. When pyrethroids are applied in other formulations (Sprays) at doses that are toxic for mosquitoes, itching, pricking sensations, numbness, burning of the skin and the eyes, or tingling of the skin are the symptoms most commonly reported after contact with pyrethroids (6 studies cited in WHO 2005). Symptoms usually start 1–6 h after exposure (3 studies cited in WHO 2005) and last not more than 24 h, in some cases they may last up to 3 days. Pyrethroids are not carcinogenic, genotoxic or toxic to reproduction in experimental animals. When WHO evaluated different uses of these AIs for public health purposes, it was reported that the onset of parasethesis and upper respiratory tract sensory irritation, especially in asthmatics cannot be ruled out but no long-term or serious health risks are anticipated from pyrethroids used at WHO recommended levels. It should be noted that the formulation used in the study is doses significantly lower than for pyrethroid spray formulations and, therefore, the likelihood of these symptoms are significantly lower*.* No special recommendations are needed for pregnant woman or children

Study staff will place products in HHs and ensure they are out of reach of children. Homeowners will be advised that products should not be touched by HH members once products have been applied. Although label instructions advise not to touch the products and keep them out of reach of children, consequences of exposure or ingestion of the cards are considered minimal; some people may experience coughing, sneezing, and eye irritation and may also have some skin sensitivity to the insecticide. This is likely to be only mild and temporary. Any health-related problems would be reported to a physician at a local health center and we will provide contact information so that a project physician could visit any participant with symptoms.

No psychological, social, or legal consequences are anticipated for this study. Participation in this study will not affect residents’ normal access to medical care and treatment through the local health department, or access to vector control administered by the Ministry of Health. Risks will be outlined during consent to house residents. For other study procedures such as the entomological surveys, questionnaires, or surveillance visits, the only potential immediate risks are as follows: Participants may find that the entomological visits, questionnaires, are inconvenient or take up too much of their time.

Of particular importance to this protocol is a clear explanation that the product being applied in their house may or may not be treated and that study personnel will not know the treatment of the product (Mosquito Shield™ or placebo). We will explain why it is necessary to have control house houses to test the product. For all studies, we will emphasize that individuals are not obligated in any way to participate and can retire from the project at any time without repercussions.

### Benefits to study participants

HHs who participate will potentially benefit from application of mosquito control measures. Repellent applications should supplement the national control program and are likely to result in a decrease of mosquito biting activity and a reduction in DENV transmission. Subjects with febrile illness will receive appropriate case management from the onset of symptoms, per the national guidelines. Should a study physician determine that the subject is suffering from severe dengue using WHO standard definitions [13] or that, even in the absence of criteria for severe dengue, the subject should be hospitalized, the subject will be referred to the local hospital and treated according to the medical judgment of the local physician. Longitudinal cohort children will be reimbursed 1500 LKR to offset the cost of coming to the clinic for each scheduled or any clinic visits. Transport to hospital, if needed by participants, will be facilitated through transport reimbursement. Hospital fees will not be paid by the study unless the illness or injury is due to study product or procedures. If there is illness or injury due to study product or procedures, the participant will be given medical care for that injury, at no cost to the participant. The participant will not receive any injury compensation, only medical care. Transport to hospital, if needed by participants, will be facilitated through transport reimbursement.

Local health staff will be involved during the study, and their skills will be strengthened in research. The competencies enhanced during the study will benefit local communities at the end of the project, as local staff health and materials will remain at their disposition.

### Frequency and plans for auditing trial conduct {23}

fhiClinical will be responsible for conducting clinical monitoring at the protocol implementation level; ensuring that subjects are properly consented; data are appropriately gathered; safety events are documented and reported as required; investigational product is stored, distributed, and collected per specifications; and trial close-out activities occur on a timely basis.

### Plans for communicating important protocol amendments to relevant parties (e.g., trial participants, ethical committees) {25}

Protocol amendments will be submitted to the Study Sponsor IRBs, local IRBs, and the WHO Ethical Review Committee (ERC) for approvals. Any amendments proposed to study activities, outcomes, analyses or more, will be communicated among investigative teams, study staff, other study stakeholders, and/or in-country public health officials. Stakeholder engagement will occur as needed to review and assess study progress and issues pertinent to further execution of the trial. Due to the potential variability in geographic location of study team affiliations, amendment discussion meetings may occur in person and/or via teleconferences.

### Dissemination plans {31a}

In order to disseminate overall study findings to participants and community members, in-country meetings will be convened with civil society members, religious leaders, and key beneficiaries. We will use a variety of community-based channels to include local newspapers, local radio stations, bulletin boards, and posters. We will also use community-based activities, such as health fairs, concerts, rallies, and parades, and community mobilization efforts organized by leaders in civil society around topics of prevention of vector-borne disease. The results of the study will be published in scientific, peer-reviewed journals and may be presented in the form of oral or poster presentations at national or international scientific meetings; however, the data and results will be presented so participant anonymity and confidentiality will be maintained.

All participants providing a blood sample receive a small written report presenting and explaining their results. With the exception of children where results are presented in the presence of parents, results will be provided to individual participants. In the case that the person is negative in the acute sample, we will emphasize we cannot know if they have had dengue until testing a convalescent sample and clinical follow-up is important. As for longitudinal sample results, we will provide a summary interpreting their collective laboratory results: infections they had previous to, and then during, the trial, based on their laboratory test results. We will explain they might be infected with DENV again. Nurse supervisors and physicians often carry out this exercise separately to reinforce the significance of results. A key message will be many people with dengue do not have obvious disease and thus do not seek treatment. Increasing awareness of dengue infection and illness is an important objective of the study. Because of inherent difficulties, cross-reactions, and variability in laboratory assays, we will emphasize this and how important trials like the one we will carry out are to understanding the value of laboratory assays.

Reports on aggregated data, especially *Ae. aegypti* indices, will be provided to the MOH in a timely manner to aid with ongoing city surveillance and control activities. This data will not contain any identifiers relating to the individual HHs or participants involved in the study; i.e., the entomological indices are calculated with reference to a certain cluster. Trial outcomes will be submitted to the WHO VCAG for use in assessing public health value of the SR product class to endorse a global policy for SR vector control. If the SR product is effective, these products may be deployed in other *Aedes*-borne disease settings to complement other interventions to enhance the reduction of dengue. Information from these studies will guide the Ministry of Health in their selection of dengue control strategies. Without the type of data generated from this trial, public health officials are unable to confidently justify the use of SRs as a new, efficacious, intervention product.

## Discussion

*Aedes*-borne viruses (ABVs) represent a broad range of pathogens of human health interest. For example, each year over 3.9 billion people in 129 countries are at risk of DENV infection [1]. As the scope of dengue continues to grow and other ABVs such as CHKV, ZIKV, Yellow Fever Virus, and Mayaro virus remain health threats, new tools are needed to compliment the limited number of available interventions and to optimize application of current products in order to meet public health demands. Additional tools, such as SRs, may address these gaps in coverage and further reduce disease burden. AIs in SR products operate through a different mode of action and have behavioral effects against insecticide-susceptible and insecticide-resistant vectors responsible for transmitting multiple human pathogens [57]. Due to their ease of implementation, SRs may help address the challenges in intervention coverage inside homes in modern urban environments. In addition, SRs may be a helpful addition in areas where insecticide resistance limits the effectiveness of other vector control tools by reducing selection pressure for insecticide resistance and thereby maintaining their natural life span [58].

There are thousands of registered SR products already available in market, adopted and used for protection from nuisance biting insects; however, there is presently no public sector use SR products for disease control due to insufficient evidence for a WHO policy recommendation. Over the past decade, national and international meetings have convened academics, industry, funders, global public health experts, and WHO representatives to discuss the role of SR products in the reduction of arthropod-borne diseases based on existing evidence.

A SR vector control product class is currently under WHO assessment for public health value. The biggest evidence gap in completing this process is the lack of epidemiological data needed to demonstrate public health impact across a range of ecological and epidemiological settings, which is needed to inform a potential WHO policy recommendation for the incorporation of SR products into current multi-lateral disease control programs. A recent large-scale clinical trial in Iquitos, Peru, using a passive transfluthrin emanator, demonstrated a significant, and conclusive, protection effect (34%) against ABV infection in trial clusters receiving the active intervention compared to placebo. The trial also detected a significant reduction in adult *Ae. aegypti* female indoor abundance (28%) and blood fed rate (12%) compared to baseline [4]. A cRCT in Sri Lanka will provide evidence from a second epidemiological trial required by the WHO VCAG to fully assess the public health value of SRs.

If the WHO VCAG endorses a policy recommendation for the SR class to be recommended for public health use, national disease control programs will have the option to adopt a SR policy and “next-in-kind” SRs (e.g., volatile pyrethroids such as metofluthrin) can be marketed in the public health channel without the need to undergo further WHO VCAG epidemiological assessment, incentivizing SR product research. Outputs will align with other global health stakeholders addressing ABV transmission prevention, insecticide resistance management, and product access and barriers to market introduction of new vector control products. If effective, SRs can be deployed to complement other vector control interventions to further reduce the burden of ABV.

### Trial status

Under protocol version 9.1 from February 3, 2022. Recruitment, screening, and enrolment of subjects for follow-up with intervention is anticipated to commence in 2023.

## Data Availability

The statistical analysis plan and analytic code will be made open access. The data and supporting information will be made available 12 months following completion of data analysis and will remain open access in the public domain. Open access repository distributed under the terms of the Creative Commons Attribution (CC-BY) License, which permits unrestricted use, distribution, and reproduction in any medium, provided the original author and source are credited.

## Abbreviations

ABV: *Aedes*-borne virus
AE: Adverse event
AEGIS: Advancing Evidence for the Global Implementation of Spatial Repellent
AI: Active ingredient
CHIKV: Chikungunya virus
CDC: Centers for Disease Control and Prevention
CPD: Critical path of development
CRC: Center for Research Computing
cRCT: Cluster-randomized control trial
DE: Diversionary effect
DENV: Dengue virus
DHF: Dengue hemorrhagic fever
DSMB: Data safety monitoring board
DSS: Dengue shock syndrome
EMEM: Eagle’s Minimum Essential Medium
ERC: Ethical Review Committee
FBS: Fetal bovine serum
FDR: False discovery rate
GCP: Good Clinical Practice
HIN: Household Identification Number
HH: Household
HMAF: Hyperimmune mouse ascitic fluid
HRP: Horseradish peroxidase
iELISA: Indirect enzyme-linked immunosorbent assay
IRB: Institutional Review Board
ITT: Intention to treat
LTFU: Loss to follow-up
MNT: Microneutralization test
MOH: Medical Officer of Health
NDCU: National Dengue Control Unit
OR: Odds ratio
PBS: Phosphate-buffered saline
PE: Protective efficacy
PI: Principal Investigator
PP: Per protocol
PRNT: Plaque Reduction Neutralization Test
RT-PCR: Reverse transcription polymerase chain reaction
SAE: Serious adverse event
SCJ: S.C. Johnson, Inc., A Family Company
SOP: Standard operating procedure
SR: Spatial repellent
UIC: Unique Identifying Code
UND: University of Notre Dame
VCAG: Vector Control Advisory Group
WHO: World Health Organization
ZIKV: Zika virus
